# Computational modeling of progressive damage and rupture in fibrous biological tissues: application to aortic dissection

**DOI:** 10.1007/s10237-019-01164-y

**Published:** 2019-05-15

**Authors:** Osman Gültekin, Sandra Priska Hager, Hüsnü Dal, Gerhard A. Holzapfel

**Affiliations:** 1 Institute of Biomechanics, Graz University of Technology, Stremayrgasse 16/II, 8010 Graz, Austria; 2 Department of Mechanical Engineering, Middle East Technical University, Dumlupınar Bulvarı No. 1, Çankaya, 06800 Ankara, Turkey; 3 Department of Structural Engineering, Norwegian University of Science and Technology (NTNU), 7491 Trondheim, Norway

**Keywords:** Fibrous biological tissues, Aortic dissection, Crack phase-field, Damage, Rupture

## Abstract

This study analyzes the lethal clinical condition of aortic dissections from a numerical point of view. On the basis of previous contributions by Gültekin et al. (Comput Methods Appl Mech Eng 312:542–566, 2016 and 331:23–52, 2018), we apply a holistic geometrical approach to fracture, namely the crack phase-field, which inherits the intrinsic features of gradient damage and variational fracture mechanics. The continuum framework captures anisotropy, is thermodynamically consistent and is based on finite strains. The balance of linear momentum and the crack evolution equation govern the coupled mechanical and phase-field problem. The solution scheme features the robust one-pass operator-splitting algorithm upon temporal and spatial discretizations. Based on experimental data of diseased human thoracic aortic samples, the elastic material parameters are identified followed by a sensitivity analysis of the anisotropic phase-field model. Finally, we simulate an incipient propagation of an aortic dissection within a multi-layered segment of a thoracic aorta that involves a prescribed initial tear. The finite element results demonstrate a severe damage zone around the initial tear and exhibit a rather helical crack pattern, which aligns with the fiber orientation. It is hoped that the current contribution can provide some directions for further investigations of this disease.

## Introduction

Aortic dissection is a lethal condition characterized by delamination and separation of adjacent lamellae within the media of the aorta. The annual incidence of aortic dissection ranges from 2 to 9 per 100,000 patients according to Clouse et al. (2004) and Howard et al. (2013). It is of utmost importance to better understand the underlying mechanisms contributing to the development of the disease. Toward this understanding, mathematically robust and physically relevant computational models can identify certain aspects of this intricate phenomenon.

### Histology of the aortic wall

**Fig. 1:**
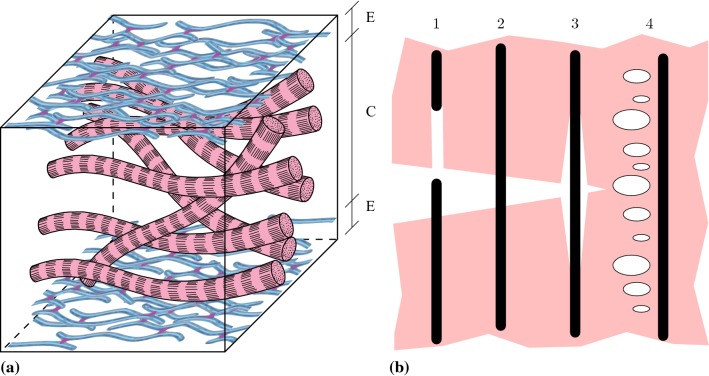
**a** Schematic view of an aortic medial lamellar unit composed of two adjacent elastic lamellae (at the top and bottom represented by the letter E) including elastin and fibrillin microfibrils. In between the elastic lamellae is the embedded collagenous lamella (denoted by the letter C) that involves smooth muscle cells, adhesion molecules as well as GAGs and PGs not shown here due to illustrative reasons. This unit corresponds to the micro-scale outlook of the aortic wall with the typical thickness of the elastic lamella E, about 1.5 $\upmu$m, and the collagenous lamella C, about $12\,\upmu \hbox {m}$, reconstructed from Ross and Pawlina (2011); **b** possible damage and fracture mechanisms that are likely to occur in the aorta during *mode I*, *mode II* and *mixed-mode* fracture. 1: collagen fiber pull-out; 2: collagen fiber bridging; 3: collagen fiber/matrix debonding; 4: matrix cracking; reconstructed from Anderson (2005)

The aorta, the main conduit for blood delivery, is an elastic artery which consists of three distinct layers: the *tunica intima*, the *tunica media* and the *tunica adventitia*. The intima is the main layer involved in metabolic processes and is comprised of mono-layered endothelial cells supported by loose connective tissue. The intima provides a negligible mechanical contribution to the wall resistance in young and healthy individuals. Separated from the intima by the internal elastic lamina is the media, which supports the aortic wall against the physiological blood pressure. The media is composed of as many as 70 fenestrated medial lamellar units, hosting two adjacent elastic lamellae (involving elastin), which are interspersed with collagen fibers, smooth muscle cells, glycosaminoglycans (GAGs) and proteoglycans (PGs), as illustrated in Fig. 1a. As reported by Schriefl et al. (2012) the media mainly involves two families of collagen fibers organized successively in the stacked laminar units, each of which contains a single fiber family. The outermost layer of the artery is the adventitia, which prevents the excessive expansion of the wall during systole in a cardiac cycle. In contrast to the media, the adventitia is formed of a network of collagen fibers arranged in a helical structure and is in general stiffer than the media (Gasser et al. 2006; Ross and Pawlina 2011; Humphrey 2013).

A closer look into the structural features of the media and the adventitia shows that the elastin and the collagen fibers are the most significant extra-cellular matrix proteins, as illustrated in Fig. 1a. Out of several damage and fracture mechanisms that occur in a variety of materials, collagen fiber pull-out, collagen fiber bridging, collagen fiber/matrix debonding and matrix cracking seem likely to occur in the aortic wall in *mode I*, *mode II* and *mixed-mode* fracture, as illustrated in Fig. 1b. Such a hypothesis is justified by some studies summarized in Sects. 1.3 and 1.4. That the ultimate rupture stress values for single human collagen fibrils generally reach hundreds of MPa s (see, e.g., Svensson et al. 2013), which is further evidence substantiating our theory that during the rupture of an aortic wall, the fiber-matrix and/or matrix–matrix interactions keeping the wall in register are disrupted, i.e., fibers themselves do not break.

### General aspects of aortic dissection

An aortic dissection may appear as an acute or a chronic type, depending on the duration of clinical symptoms (Criado 2011). Of all factors that can be associated with an aortic dissection, hypertension appears to be the most common predisposing factor, diagnosed in 77% of patients (Mussa et al. 2016). Among other factors are the cystic medial necrosis, connective tissue disorders, e.g., Marfan syndrome and Ehlers–Danlos syndrome, aneurysm, trauma, e.g., car accidents, cardiac catheterization, male sex, and individuals aged 60 to 70 years (Dunning et al. 2000; Criado 2011; Tsamis et al. 2013).

**Fig. 2:**
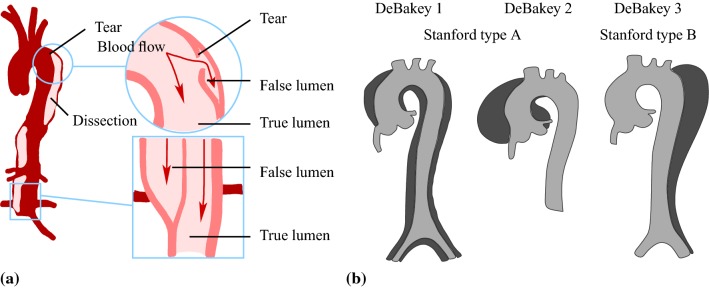
**a** Schematic view of an aortic dissection which starts with an initial tear near the left subclavian artery and propagates downwards, while following a path that results in the formation of a false lumen next to the true one; **b** classifications of the aortic dissection: Stanford type A involves the ascending aorta and Stanford type B excludes the ascending part. In the DeBakey system, type 1 includes the entire aorta, type 2 contains only the ascending aorta, and type 3 excludes the ascending aorta, reconstructed from Tsamis et al. (2013)

In most cases the initial phase of an aortic dissection manifests itself as an initial tear in the intima due to some structural weakening in a localized part of the endothelium, see Fig. 2a, the cause of which is rather elusive. An aortic dissection, however, may also commence within the wall due to an intramural hemorrhage or a hematoma formation in the media (Thubrikar et al. 1999; Khan and Nair 2002). Two-thirds of all initial tears happen within the ascending aorta at about 2 cm above the aortic root near the sinotubular junction (Pasta et al. 2012). The second common location is the isthmus of the descending thoracic aorta located beyond the origin of the left subclavian artery, as depicted in Fig. 2a (Cherry and Dake 2009). From the hemodynamics point of view, the most likely reason that makes these areas prone to aortic dissection is the turbulent pulsatile blood flow imposed by the geometry, i.e., the blood pumped out of the left ventricle has to navigate the turn of the aortic arch (Rajagopal et al. 2007; Cherry and Dake 2009). Upon the initial tear formation, which may be seen as a radial propagation across the sub-layers of the media, the dissection changes its course and follows a rather helical path. The dissection separates the lamellar units and creates a false lumen, see Fig. 2a. In any case, a crack propagation across the entire lamellae is not energetically favored (Ottani et al. 2001). A significant amount of the blood now enters the dissected part of the wall, causing even more tearing as blood jets through the tear in each cardiac cycle. Thereafter, the dissection may continue to propagate down toward the abdominal aorta or create an exit (reentry) tear so that the blood can flow back into the true lumen (Cherry and Dake 2009). In the absence of an exit tear, more and more blood enters the false lumen, which decreases the blood volume through the true lumen, leaving the remaining wall susceptible to a dilatation. Over time this may cause other severe pathologies such as the rupture of the remaining aortic wall.

Aortic dissections are classified according to the Stanford system and the DeBakey system, as depicted in Fig. 2b. In the Stanford classification, type A involves the ascending segment of the aorta, whereas in type B the ascending aorta is not affected. The DeBakey system, on the other hand, characterizes three different types of aortic dissection: type 1 involves the ascending and descending part of the aorta, and commonly extends beyond the arch distally; in type 2 the only part affected is the ascending aorta; type 3 describes an aortic dissection excluding the ascending part (Tsamis et al. 2013).

### Experimental studies on aortic dissection

Early experimental investigations on aortic dissections have mostly followed methods such as pressurization, direct tension and peel tests. Carson and Roach (1990) and Roach and Song (1994) infused fluid via a needle through the media extracted from porcine aortas, causing a bleb formation starting at a non-physiological pressure value of 579 mmHg. Notably, the dissection generated continued to propagate at physiological pressure values. In another study by Tam et al. (1998), the pressure was found to be inversely correlated with the initial tear depth under static conditions. All studies stated above reported on the energy release rates (mJ/cm$^2$), i.e., the energy expended to create a unit area of ruptured material, a significant concept in fracture mechanics. Later the experimental studies focused more on the direct tension and peel tests, which were presented by Sommer et al. (2008), Tong et al. (2011), Pasta et al. (2012) and Wang et al. (2014), among others. All the investigations provided energy release rates, which were found to vary over the different aortic layers and along the different orientations for which the tests were conducted, i.e., circumferential and longitudinal directions.

Microscopical images of the dissected surfaces in the study of Sommer et al. (2008) provided visual evidences for the fiber pull-out and fiber/matrix debonding mechanisms. Nonetheless, direct tension, peel and trouser tests led to a delamination of the adjacent medial lamellae, which is undoubtedly driven by normal stresses ($\sigma _{rr}$, $\sigma _{\theta \theta }$, $\sigma _{zz}$), i.e., *mode I* fracture. Later, Sommer et al. (2016) found that ultimate in-plane shear stresses ($\sigma ^u_{r\theta }$, $\sigma ^u_{rz}$) are about an order of magnitude lower than ultimate out-of-plane shear stresses ($\sigma ^u_{\theta z}$), a result which is based on simple shear tests performed on medial specimens cut from the human thoracic aortas. This result can be attributed to the collagen fibers embedded in the ground matrix that are barely deformed in their mean orientation in the case of in-plane shear tests. Hence, the aortic dissection is less likely to propagate in the radial direction running across several lamellae, a conclusion which was also proposed by Haslach et al. (2018). The authors of Haslach et al. (2018) concluded that the dissection propagation, which can be explained by the relative slip between the adjacent medial lamellae in the circumferential–longitudinal plane, is an in-plane shear driven process evoking *mode II* fracture.

### Numerical studies on aortic dissection

To date, aortic dissection has been the subject matter of several numerical models in solid mechanics. Ferrara and Pandolfi (2010) applied a cohesive zone model (CZM) together with an anisotropic traction-separation law and investigated one of the peel tests conducted by Sommer et al. (2008). In an experimental and computational study, Leng et al. (2018) employed the CZM to fit the model parameters to load–displacement curves obtained from a shear dominated *mixed-mode* and a *mode I* peel test along both circumferential and longitudinal directions. Apart from that, Noble et al. (2017) carried out experimental and computational analyses of a catheter-induced delamination. By using the partition of unity finite element method, Gasser and Holzapfel (2006) studied peel tests. Other studies, adopting the extended finite element method (XFEM), were presented by Wang et al. (2017, 2018) where peel tests similar to the aforementioned contributions were examined numerically. Moreover, the authors therein presented an inflation test of a residually stressed plane strain solid model of a hollow circle representing the cross section of an aortic wall, with varying opening angles. An identical blood pressure was applied on both the inner layer and the tear edge. The tear edge describes a prescribed circumferentially dissected zone where the nodal enrichments are introduced. As a result, the critical pressure value for initiation of aortic dissection propagation was found to increase with the opening angle; therefore, residual stresses seem to protect the artery from tear propagation. Even though several simplified models (mostly 2-D) exist using CZM and XFEM, which are relatively easy to handle in terms of tracking the discontinuities by means of remeshing and nodal/element enrichment functions, more complex and histologically representative 3-D geometries lead to an arduous task when CZM and XFEM are used.

In contrast, the crack phase-field approach (CPFA) by Francfort and Marigo (1998) circumvents the modeling of discontinuities (Miehe et al. 2015a, 2016; Ambati et al. 2015; Borden et al. 2016). Resembling the gradient damage models, CPFA contains the critical fracture energy $g_{\mathrm{c}}$ (critical energy release rate), an essential ingredient of fracture mechanics. Within CPFA, Gültekin et al. (2016) and Gültekin and Holzapfel (2018) studied rupture in human aortic tissues induced by uniaxial extension, simple shear and peeling. They assumed that distinct fracture mechanisms were involved in the overall macroscopic fracture process and they introduced separate critical fracture energies, $g_{\mathrm{c}}^{\mathrm{iso}}$ for the ground matrix, and $g_{\mathrm{c}}^{\mathrm{ani}}$ for the fibrous content. These represent the up-scaled, homogenized, macro-resistance of the interactions within the ground matrix and between the matrix and collagen fibers against damage and rupture. A more elaborate account of the extant computational solid models can be found in Gültekin et al. (2018).

Aside from the benchmark solid models presented in the literature, there are a few patient-specific studies dealing with computational fluid dynamics (CFD) for exploring the underlying hemodynamics of the aorta. Tse et al. (2011) found a blood pressure difference as high as 210 Pa between the true and the false lumen which pinpointed the proximal ascending aorta and the distal aortic arch as the areas subject to the vortex flow, these coinciding with the prevalent initial tear locations, see Sect. 1.2. Besides, on the basis of a helical blood pattern observed solely in the ascending aorta, the authors inferred that the clinically observed helical dissection propagation is the result of the vortex flow. Cheng et al. (2013) showed that the aortic morphology, the initial tear size and the position influence the flow and other hemodynamic parameters. Although most of the CFD studies consider the wall shear stress as a direct contributor to the development of the aortic dissection, such a hypothesis does not seem probable as the wall shear stresses range between 3 and 10 Pa, which are negligibly small compared with the stresses experienced within the wall. It is also worth mentioning some fluid–solid interaction models within the context of aortic dissection (Qiao et al. 2015; Malvindi et al. 2017).

### Aim of the present study

In view of our previous findings and contributions (Gültekin et al. 2016; Gültekin and Holzapfel 2018), the present study delivers a computational protocol with novel features for investigating the nascent aortic dissection, and addresses certain mechanical aspects of the protocol, based on the phase-field modeling of progressive damage and rupture.

The article is organized as follows. Section 2 offers a tour of the continuum mechanical and algorithmic framework in terms of geometry, kinematics, governing balance equations and the solution strategy. Section 3 outlines a parameter identification procedure for experimental data obtained from a diseased human aorta and provides a sensitivity analysis of an anisotropy parameter for the extension of a squared single-edge notched domain. In addition, certain physical aspects of an nascent aortic dissection are studied in an idealized cylindrical tube with a prescribed initial tear. Sections 4 and 5 provide a critical discussion and overview of some open problems and possible improvements regarding the modeling concept discussed herein.

## Multi-field framework for rupture

This section is devoted to anisotropic phase-field modeling of fracture. The primary field variables, namely the crack phase-field *d* and the deformation map ${\varvec{\varphi }}$, are introduced along with their governing equations, i.e., the evolution of the crack phase-field and the balance of linear momentum. Subsequently, an account on the numerical edifice is briefly given which features the operator-splitting algorithm. For the related continuum mechanics see, e.g., the books by Ogden (1997) and Holzapfel (2000).

### Primary field variables of the multi-field problem

**Fig. 3:**
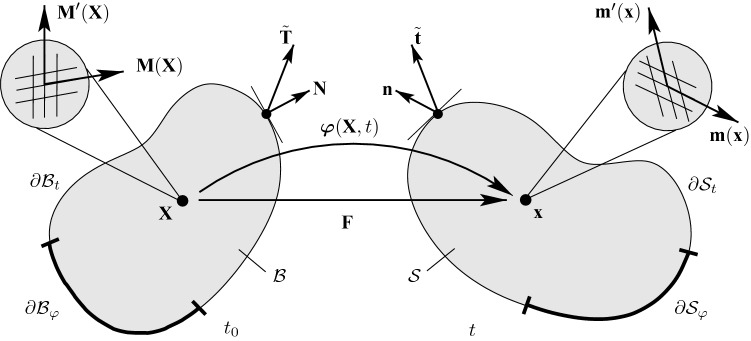
Nonlinear deformation of an anisotropic solid with the reference configuration ${\mathcal {B}}\subset {\mathbb {R}}^3$ and the spatial configuration ${\mathcal {S}}\subset {\mathbb {R}}^3$. The surface boundary associated with ${\mathcal {B}}$ is defined by $\partial {\mathcal {B}}\subset {\mathbb {R}}^2= \partial {\mathcal {B}}_\varphi \cup \partial {\mathcal {B}}_t$ and $\partial {\mathcal {B}}_\varphi \cap \partial {\mathcal {B}}_t = \emptyset$, and the respective surface boundary in ${\mathcal {S}}$ is given by $\partial {\mathcal {S}}\subset {\mathbb {R}}^2 = \partial {\mathcal {S}}_\varphi \cup \partial {\mathcal {S}}_t$ and $\partial {\mathcal {S}}_\varphi \cap \partial {\mathcal {S}}_t = \emptyset$. The surface tractions ${{\tilde{\mathbf{T}}}}$ and ${{\tilde{\mathbf{t}}}}$ are applied on $\partial {\mathcal {B}}_t$ and $\partial {\mathcal {S}}_t$ with unit normals $\mathbf{N}$ and $\mathbf{n}$ pointing outward, respectively. The map ${\varvec{\varphi }}: {\mathcal {B}}\times {\mathcal {T}}\rightarrow {\mathcal {S}}$ transforms a material point $\mathbf{X}\in {\mathcal {B}}$ onto a spatial point $\mathbf{x}= {\varvec{\varphi }}(\mathbf{X}, t) \in {\mathcal {S}}$ at time *t*. The anisotropic micro-structure of $\mathbf{X}$ is rendered by two families of fibers with unit vectors $\mathbf{M}$ and $\mathbf{M}^\prime$. Likewise, the anisotropic micro-structure of $\mathbf{x}$ is described by $\mathbf{m}$ and $\mathbf{m}^\prime$ as the spatial counterparts of $\mathbf{M}$ and $\mathbf{M}^\prime$, respectively

**Fig. 4:**
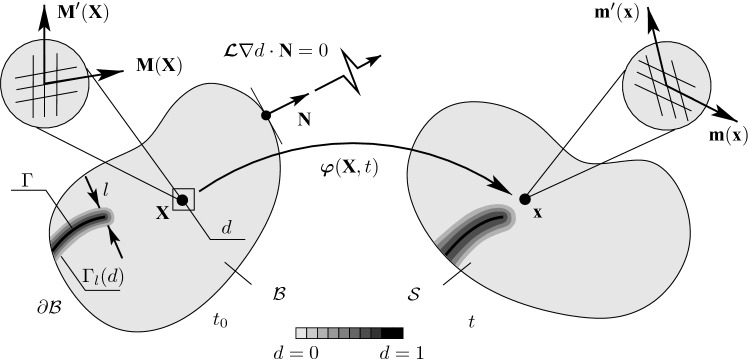
Schematic view of the diffusive crack topology in ${\mathcal {B}}$ and ${\mathcal {S}}$ with the crack phase-field $d: {\mathcal {B}}\times {\mathcal {T}}\rightarrow [0,1]$, while the sharp crack surface $\Gamma$ smears out in the respective solid domain, denoted by $\Gamma _l(d)$, which is regularized by the length scale parameter *l*. The material anisotropy is imparted by the two families of fibers with the material unit vectors $\mathbf{M}$ and $\mathbf{M}^\prime$ along with their spatial counterparts $\mathbf{m}$ and $\mathbf{m}^\prime$, respectively. The anisotropic crack phase-field problem is further characterized by a traction-free Neumann-type boundary condition ${\varvec{\mathcal {L}}}\nabla d \cdot \mathbf{N}= 0$ on $\partial {\mathcal {B}}$

Let ${\mathcal {B}} \subset {\mathbb {R}}^3$ be a continuum body at time $t_0 \in {\mathcal {T}}\subset {\mathbb {R}}^+$ and ${\mathcal {S}} \subset {\mathbb {R}}^3$ at current time $t \in {\mathcal {T}}\subset {\mathbb {R}}^+$ in the Euclidean space. The coupled problem of rupture is expressed by the bijective deformation map ${\varvec{\varphi }}_t$ and the auxiliary crack phase-field *d*, i.e.,

1 $$\begin{aligned}&{\varvec{\varphi }}_t(\mathbf{X}) : {\left\{ \begin{array}{ll} {\mathcal {B}}\times {\mathcal {T}}&{} \rightarrow \quad {\mathcal {S}}, \\ (\mathbf{X}, t) &{} \mapsto \quad \mathbf{x}= {\varvec{\varphi }}(\mathbf{X},t), \end{array}\right. } \nonumber \\&d: {\left\{ \begin{array}{ll} {\mathcal {B}}\times {\mathcal {T}}&{} \rightarrow \quad [0,1], \\ (\mathbf{X},t) &{} \mapsto \quad d(\mathbf{X},t), \end{array}\right. } \end{aligned}$$

where ${\varvec{\varphi }}_t$ maps a material point $\mathbf{X}\in {\mathcal {B}}$ onto a spatial point $\mathbf{x}\in {\mathcal {S}}$, see Fig. 3, while *d*, a thermodynamic measure of damage in the solid, interpolates between the intact ($d=0$) and the ruptured ($d=1$) state of the material corresponding to a domain regulated by the length scale parameter *l*, as illustrated in Fig. 4. Note that the crack phase-field *d* is formulated in the reference configuration ${\mathcal {B}}$.

### Kinematics of the mechanical problem

Let the gradient operators $\nabla (\bullet )$ and $\nabla _x(\bullet )$ denote the gradients with respect to the reference and the spatial coordinates $\mathbf{X}$ and $\mathbf{x}$, respectively. The continuous manifolds are locally equipped with the covariant reference and spatial metric tensors $\mathbf{G}= \delta _{IJ} \mathbf{E}^{I} \otimes \mathbf{E}^{J}$ and $\mathbf{g}= \delta _{ij} \mathbf{e}^{i} \otimes \mathbf{e}^{j}$, respectively, where $\delta _{IJ}$ and $\delta _{ij}$ denote the Kronecker deltas. The bodies ${\mathcal {B}}$ and ${\mathcal {S}}$ admit the deformation gradient $\mathbf{F}$ and the left Cauchy–Green tensor $\mathbf{b}$ such that

2 $$\begin{aligned} \displaystyle \mathbf{F}= \nabla {\varvec{\varphi }}\quad \text {and} \quad \mathbf{b}= \mathbf{F}\mathbf{G}^{-1} \mathbf{F}^{\mathrm{T}}, \end{aligned}$$

for non-penetrable deformations, i.e., $J>0$, where $J=\text {det}\mathbf{F}$, see Fig. 3. The energy stored in a hyperelastic isotropic continuum is characterized by the three independent invariants

3 $$\begin{aligned} \displaystyle I_1 = \mathrm{tr}\mathbf{b}, \quad I_2 = \frac{1}{2}\left[ I_1^2-\mathrm{tr}(\mathbf{b}^2)\right] , \quad I_3 = \det \mathbf{b}. \end{aligned}$$

The anisotropic structure of the aortic wall is described by two reference unit vectors $\mathbf{M}$ and $\mathbf{M}^\prime$ representing the mean fiber orientations, see Fig. 3, with their spatial counterparts, i.e., $\mathbf{m}= \mathbf{F}\mathbf{M}$ and $\mathbf{m}^\prime = \mathbf{F}\mathbf{M}^\prime$. This idealization of the tissue micro-structure leads to the respective Eulerian form of the structure tensors, i.e.,

4 $$\begin{aligned} \displaystyle \mathbf{A}_{{{\mathbf{m}}}} = \mathbf{m}\otimes \mathbf{m}, \quad \mathbf{A}_{{{\mathbf{m}}}^\prime } = \mathbf{m}^\prime \otimes \mathbf{m}^\prime , \end{aligned}$$

with their Lagrangean counterparts, i.e., $\mathbf{A}_{{{\mathbf{M}}}} = \mathbf{M}\otimes \mathbf{M}$ and $\mathbf{A}_{{{\mathbf{M}}}^\prime } = \mathbf{M}^\prime \otimes \mathbf{M}^\prime$. Next we introduce the (physically meaningful) additional invariants

5 $$\begin{aligned} \displaystyle I_4 = \mathbf{m}\cdot \mathbf{g}\mathbf{m}, \quad I_6 = \mathbf{m}^\prime \cdot \mathbf{g}\mathbf{m}^\prime , \end{aligned}$$

which measure the squares of stretches along each mean fiber direction.

### Kinematics of the phase-field problem

Let $\Gamma$ be a discontinuous boundary such that $\Gamma \in \partial {\mathcal {B}}_{\varphi } \subset {\mathbb {R}}^{2}$ at time $t_0$, which characterizes a sharp crack surface, i.e., $\Gamma = \int _{\Gamma }\mathrm{d}A$, as indicated by a thick solid curve in Fig. 4. Instead of tracking such an interface, the phase-field approach approximates the surface integral by a volume integral engendering a regularized crack surface $\Gamma _{l} (d)$, i.e.,

6 $$\begin{aligned}&\displaystyle \Gamma _{l}(d) = \int \limits _{{\mathcal {B}}}\gamma (d,\nabla d) \mathrm{d}V, \quad \text {where} \nonumber \\&\displaystyle \gamma (d,\nabla d) = \frac{1}{2l}(d^2 + l^2 \nabla d \cdot \nabla d) \end{aligned}$$

describes the *isotropic crack surface density* function that satisfies the condition $\gamma (d, \mathbf{Q}\nabla d) = \gamma (d, \nabla d)$, $\forall \mathbf{Q}\in {\mathcal {O}}(3)$. The tensor variable $\mathbf{Q}$ denotes the rotations in the orthogonal group ${\mathcal {O}}(3)$, which contains rotations and reflections. This approximation can be extended to a class of anisotropic materials such that

7 $$\begin{aligned}&\displaystyle \Gamma _{l}(d) = \int \limits _{{\mathcal {B}}}\gamma (d,\nabla d;\, {\varvec{\mathcal {L}}}) \mathrm{d}V, \quad \text {where} \nonumber \\&\displaystyle \gamma (d,\nabla d;\, {\varvec{\mathcal {L}}}) = \frac{1}{2l}(d^2 + \nabla d \cdot {\varvec{\mathcal {L}}}\nabla d) \end{aligned}$$

describes the *anisotropic crack surface density* function subject to the condition $\gamma (d, \mathbf{Q}\nabla d) = \gamma (d, \nabla d)$, $\forall \mathbf{Q}\in {\mathcal {G}}\subset {\mathcal {O}}(3)$, in which ${\mathcal {G}}$ designates a symmetry group as a subset of ${\mathcal {O}}(3)$. In (7), the second-order anisotropic structure tensor ${\varvec{\mathcal {L}}}$ reads

8 $$\begin{aligned} {\varvec{\mathcal {L}}}= l^2(\mathbf{I}+ \omega _{\mathrm{M}} \mathbf{M}\otimes \mathbf{M}+ \omega _{{\mathrm{M}}^{\prime }} \mathbf{M}^{\prime } \otimes \mathbf{M}^{\prime }), \end{aligned}$$

which aligns the crack with the orientation of fibers in the continuum as the phase-field evolves. Therein, the anisotropy parameters $\omega _{\mathrm{M}}$ and $\omega _{{\mathrm{M}}^{\prime }}$ regulate the transition from weak to strong anisotropy for two families of fibers. Hence, in the limit case, i.e., $\omega _i \rightarrow \infty$, $i \in \{{M}, M^{\prime } \}$, the crack path perfectly lies parallel to the fiber directions. For isotropic solids, the parameters $\omega _{\mathrm{M}} = \omega _{{\mathrm{M}}^{\prime }}$ are zero, whereas for a general anisotropic continuum with several families of fibers, each of them lying in an open range, i.e., $-1<\omega _i < \infty$, $i \in \{{M}, M^{\prime }, \ldots \}$, dictated by the ellipticity condition for $\Gamma _{l}(d)$ (Teichtmeister et al. 2017; Gültekin and Holzapfel 2018).

### Euler–Lagrange equations of the phase-field problem

In view of (7), we can state the minimization principle for the regularized crack surface $\Gamma _l(d)$ as

9 $$\begin{aligned} d(\mathbf{X}, t) = \text{ Arg } \begin{Bmatrix} \underset{d \in {\mathcal {W}}_{\Gamma (t)}}{\text{ inf }}\Gamma _{l}(d) \end{Bmatrix}, \end{aligned}$$

subject to the Dirichlet-type boundary constraint

10 $$\begin{aligned} \displaystyle {\mathcal {W}}_{\Gamma (t)} = \{ d | d(\mathbf{X},t) = 1 \quad \text{ at } \quad \mathbf{X}\in \Gamma (t)\}. \end{aligned}$$

Upon the minimization of the regularized crack surface functional, we derive the Euler–Lagrange equations, i.e.,

11 $$\begin{aligned}&\displaystyle \frac{1}{l} [ d - \mathrm{Div}( {\varvec{\mathcal {L}}}\nabla d) ] = 0 \quad \text{ in } \,\, {\mathcal {B}}, \quad \text{ and } \nonumber \\&{\varvec{\mathcal {L}}}\nabla d \cdot \mathbf{N}= 0 \quad \text{ on } \,\, \partial {\mathcal {B}}, \end{aligned}$$

where the divergence term interpolates *d* between the intact and the ruptured state of the material, while $\mathbf{N}$ denotes the unit surface normal pointing outwards in the reference configuration.

### A particular form of the degradation function

The macroscopic damage accumulated in the anisotropic solid manifests itself in the mechanical response in the sense of a degradation, which may assume generic distinct functional forms in accordance with the additively split isotropic and anisotropic mechanical contributions, i.e.,

12 $$\begin{aligned} \displaystyle g_{\mathrm{iso}}(d) = (1-d)^{a_{\mathrm{iso}}}, \quad \quad g_{\mathrm{ani}}(d) = (1-d)^{a_{\mathrm{ani}}}, \end{aligned}$$

satisfying the following growth conditions

13 $$\begin{aligned}&\displaystyle g^\prime _i (d) \le 0 \quad \text {with} \nonumber \\&g_i(0) =1, \quad g_i(1) = 0, \quad g^\prime _i(1) = 0, \end{aligned}$$

where $i \in \{\text{iso},\ \text{ani} \}$. The first condition ensures monotonic degradation, while the second and third conditions set the limits for the intact and the ruptured states, and the final condition ensures the saturation of $g_i(d)$ as $d\rightarrow 1$ provided that $a_{\mathrm{iso}}$ and $a_{\mathrm{ani}}$, which control the rate of the mechanical degradation with respect to the evolution of *d*, lie in the open range $(a_{\mathrm{iso}}, a_{\mathrm{ani}}) \in (1, \infty )$. We further emphasize that $a_{\mathrm{iso}}$ and $a_\mathrm{ani}$ can also be functions of stretch or stress and identified via *ex vivo* biomechanical experiments, see Sect. 4 for a discussion. For the sake of simplicity, we restrict ourselves to the quadratic degradation, i.e., $a_{\mathrm{iso}}=2$ and $a_{\mathrm{ani}} = 2$, as given in Miehe et al. (2010b), which also retrieves the multi-field formulation of the fracture problem presented in Gültekin et al. (2016) and Gültekin and Holzapfel (2018).

### A particular form of the anisotropic constitutive model

We now briefly describe the specific form of the effective Helmholtz free-energy function for the hyperelastic anisotropic mechanical response of the aortic wall, which can be split into an isotropic and an anisotropic part, i.e.,

14 $$\begin{aligned} \Psi _0(\mathbf{g}, \mathbf{F}, J, \mathbf{A}_{{{\mathbf{m}}}}, \mathbf{A}_{{{\mathbf{m}}}^\prime })=\  & {} \Psi _0^{\mathrm{iso}}(\mathbf{g}, \mathbf{F}, J) \nonumber \\&+ \Psi _0^{\mathrm{ani}}(\mathbf{g}, \mathbf{F}, \mathbf{A}_{{{\mathbf{m}}}}, \mathbf{A}_{{{\mathbf{m}}}^\prime }), \end{aligned}$$

for which the effective isotropic and the anisotropic parts can be expressed as functions of invariants, i.e.,

15 $$\begin{aligned}&\Psi _0^{\mathrm{iso}}(\mathbf{g}, \mathbf{F}, J) = {\hat{\Psi }}_0^{\mathrm{iso}}(J, I_1), \nonumber \\&\Psi _0^{\mathrm{ani}}(\mathbf{g}, \mathbf{F}, \mathbf{A}_{{{\mathbf{m}}}}, \mathbf{A}_{{{\mathbf{m}}}^\prime }) = {\hat{\Psi }}_0^{\mathrm{ani}}( I_4, I_6). \end{aligned}$$

The effective isotropic part follows from the neo-Hookean model accounting for the mechanical response of the ground matrix, whereas the effective anisotropic response features the hyperelasticity of the two families of collagen fibers. The explicit forms of $\Psi _0^{\mathrm{iso}}$ and $\Psi _0^{\mathrm{ani}}$ are proposed in Holzapfel et al. (2000) (it is straightforward to adopt here any anisotropic model, see, e.g., Holzapfel and Ogden 2017a, b). In what follows, the mechanical response of the degrading wall due to macroscopic damage in the isotropic and the anisotropic parts is stated via the degradation function in (12) such that

16 $$\begin{aligned} \Psi (\mathbf{g}, \mathbf{F}, J, \mathbf{A}_{{{\mathbf{m}}}}, \mathbf{A}_{{{\mathbf{m}}}^\prime };d)= \ & {} g_\mathrm{iso}(d)\Psi _0^{\mathrm{iso}}(\mathbf{g}, \mathbf{F}, J) \nonumber \\&+ g_{\mathrm{ani}} (d)\Psi _0^{\mathrm{ani}}(\mathbf{g}, \mathbf{F}, \mathbf{A}_{{{\mathbf{m}}}}, \mathbf{A}_{{{\mathbf{m}}}^\prime }), \end{aligned}$$

which modifies the undamaged energy storage function given in (14). The respective expressions for the Kirchhoff stress and the elasticity tensors are presented in Gültekin and Holzapfel (2018).

### Governing equations of the anisotropic fracture

This section is devoted to the governing equations of the coupled multi-field problem of fracture where the classical balance of linear momentum is accompanied by the evolution equation for the crack phase-field, for the strong form of the boundary-value problem. More details can be found in Gültekin and Holzapfel (2018). For the canonically compact gradient-damage formulations of the boundary-value problems in regard to the standard dissipative solids, the interested reader is referred to Mielke and Roubíček (2006) and Miehe (2011).

#### Rate-dependent variational formulation based on power balance

As a point of departure, we introduce the viscous rate-type potential $\Pi _{\eta }$ as

17 $$\begin{aligned} \displaystyle \Pi _{\eta } = {\mathcal {E}}+ {\mathcal {D}}_{\eta } - {\mathcal {P}}. \end{aligned}$$

The first term ${\mathcal {E}}$ on the right-hand side of (17) represents the rate of energy storage functional, i.e.,

18 $$\begin{aligned} \displaystyle {\mathcal {E}}({{\dot{{\varvec{\varphi }}}}}; \dot{d}) = \int \limits _{{\mathcal {B}}} (\, {\varvec{\tau }}: \mathbf{g}\nabla _x{\dot{{\varvec{\varphi }}}} - f {\dot{d}} \, ) \mathrm{d}V, \end{aligned}$$

where the work conjugate variables to ${\varvec{\varphi }}$ and *d* are the Kirchhoff stress tensor ${\varvec{\tau }}$ and the scalar energetic force *f*, respectively, i.e.,

19 $$\begin{aligned}&{\varvec{\tau }}= 2 \partial _{{{\mathbf{g}}}} \Psi (\mathbf{g}, \mathbf{F}, \mathbf{A}_{{{\mathbf{m}}}}, \mathbf{A}_{{{\mathbf{m}}}^\prime };\, d), \nonumber \\&f = - \partial _{d} \Psi (\mathbf{g}, \mathbf{F}, \mathbf{A}_{{{\mathbf{m}}}}, \mathbf{A}_{{{\mathbf{m}}}^\prime };\, d). \end{aligned}$$

The second term ${\mathcal {D}}_{\eta }$ on the right-hand side of (17) is a viscous regularized dissipation functional due to fracture, i.e.,

20 $$\begin{aligned} \displaystyle {\mathcal {D}}_{\eta }({\dot{d}}, \beta ; d) = \int \limits _{{\mathcal {B}}} \left[ \beta {\dot{d}} - \frac{1}{2\eta } \langle \chi (\beta ; d, \nabla d) \rangle ^2\right] \mathrm{d}V, \end{aligned}$$

where the artificial viscosity $\eta \ge 0$ regulates the scalar viscous over-stress $\chi$ that reads

21 $$\begin{aligned} \displaystyle \chi (\beta ;\, d, \nabla d) = \beta - g_{\mathrm{c}} [\delta _d \gamma (d, \nabla d;\, {\varvec{\mathcal {L}}})] , \end{aligned}$$

for which the Macaulay brackets ($$<x>= (x + |x|)/2 $$) in (20) filter out the positive values. Note that in (21), $$g_{\mathrm{c}}$$ stands for the critical fracture energy. Finally, the last term $${\mathcal {P}}$$ on the right-hand side of (17) denotes the (classical) external power functional acting on the body according to

22 $$\begin{aligned} \displaystyle {\mathcal {P}}({{\dot{{\varvec{\varphi }}}}}) = \displaystyle \int \limits _{\mathcal {B}}\rho _0 {\tilde{{\varvec{\gamma }}}} \cdot {\dot{{\varvec{\varphi }}}}\mathrm{d}V + \int \limits _{\partial {\mathcal {B}}_t}{\tilde{\mathbf{t}}}\cdot {\dot{{\varvec{\varphi }}}}\mathrm{d}a, \end{aligned}$$

where $\rho _0$, ${\tilde{{\varvec{\gamma }}}}$ and ${\tilde{\mathbf{t}}}$ represent the material density, the prescribed spatial body force and the spatial surface traction, respectively. Now, with the rate-type potential $\Pi _{\eta }$ at hand, we propose a mixed variational principle of the evolution problem as

23 $$\begin{aligned} \displaystyle \{{\dot{{\varvec{\varphi }}}}, {\dot{d}}, \beta \} = \text{ Arg } \begin{Bmatrix} \underset{{\dot{{\varvec{\varphi }}}} \in {\mathcal {W}}_{ {{\dot{\varphi }}}}}{\text{ inf }}\, \underset{ \dot{d} \in {\mathcal {W}}_{\dot{d}}}{\text{ inf }}\, \underset{\beta \ge 0}{\text{ sup }}\, \Pi _{\eta } \end{Bmatrix}, \end{aligned}$$

with the admissible domains for the primary variables

24 $$\begin{aligned}&\displaystyle {\mathcal {W}}_{{{\dot{\varphi }}}} = \{{\dot{{\varvec{\varphi }}}} \mid {\dot{{\varvec{\varphi }}}} = \mathbf{0}\quad \text{ on } \quad \partial {\mathcal {B}}_{\varphi }\}, \nonumber \\&\displaystyle {\mathcal {W}}_{\dot{d}} = \{ {\dot{d}} \mid {\dot{d}} = 0 \quad \text{ on } \quad \partial {\mathcal {B}}_d\}. \end{aligned}$$

Afterwards, the variation of the potential $\Pi _{\eta }$ with respect to the fields $\{{{\dot{{\varvec{\varphi }}}}}, \, \dot{d}, \, \beta \}$ along with simple algebraic manipulations via elimination and substitution of the respective terms (see Gültekin and Holzapfel 2018 for more details) yields the strong form of the field equations, i.e.,

25 $$\begin{aligned} \begin{array} {r@{~~}l} 1: &{} \displaystyle J {\text{div}} (J^{-1} {\varvec{\tau }}) + \rho _0 {\tilde{{\varvec{\gamma }}}} = \mathbf{0}, \\ 2: &{} \displaystyle \eta \dot{d} = 2(1-d){\overline{{\mathcal {H}}}} - d + \mathrm{Div}( {\varvec{\mathcal {L}}}\nabla d). \end{array} \end{aligned}$$

The first equation in (25) simply describes the balance of linear momentum, whereas the latter states the evolution equation for the crack phase-field in which ${\overline{{\mathcal {H}}}}$ indicates the crack driving source term such that

26 $$\begin{aligned} {\overline{{\mathcal {H}}}} = \frac{\Psi _0}{g_{\mathrm{c}}/l}. \end{aligned}$$

#### Energy-based anisotropic failure criterion

Following Gültekin et al. (2016) and Gültekin and Holzapfel (2018), an anisotropic failure criterion is now used. We begin with the assumption that two distinct failure processes govern the cracking of the ground matrix and the fibers whereby the anisotropic structure tensor ${\varvec{\mathcal {L}}}$ in (8) is additively split into distinct forms as

27 $$\begin{aligned} \displaystyle {\varvec{\mathcal {L}}}^{\mathrm{iso}}= l^2 \mathbf{I}, \quad {\varvec{\mathcal {L}}}^\mathrm{ani}= l^2 (\omega _{\mathrm{M}}\mathbf{M}\otimes \mathbf{M}+ \omega _{{\mathrm{M}}^{\prime }} \mathbf{M}^{\prime } \otimes \mathbf{M}^{\prime }). \end{aligned}$$

We introduce now $g_{\mathrm{c}}^{\mathrm{iso}}$ and $g_{\mathrm{c}}^{\mathrm{ani}}$ corresponding to the critical fracture energies attributed to the ground matrix (isotropic) and the fibrous content (anisotropic) of the aortic wall, respectively, which homogenize the mechanical resistance of the respective interactions against rupture. Such a model consideration is suitable for the description of the mechanical response of fibrous biological tissues. The crack driving source term in (26) can therefore be decomposed as

28 $$\begin{aligned} \displaystyle {\overline{{\mathcal {H}}}}^{\mathrm{iso}} = \frac{\Psi _0^{\mathrm{iso}}}{g_{\mathrm{c}}^{\mathrm{iso}}/l}, \quad {\overline{{\mathcal {H}}}}^{\mathrm{ani}} = \frac{\Psi _0^{\mathrm{ani}}}{g_{\mathrm{c}}^{\mathrm{ani}}/l}. \end{aligned}$$

For a rate-independent case where $\eta \rightarrow 0$, the above expressions (27) and (28) in conjunction with (25)$_2$ lead to distinct evolution equations of the crack phase-field in relation to the ground matrix and the fibrous content, i.e.,

29 $$\begin{aligned}&\displaystyle 2(1-d){\overline{{\mathcal {H}}}}^{\mathrm{iso}} = \displaystyle d - \mathrm{Div}( {\varvec{\mathcal {L}}}^{\mathrm{iso}} \nabla d), \nonumber \\&\displaystyle 2(1-d){\overline{{\mathcal {H}}}}^{\mathrm{ani}} = \displaystyle d - \mathrm{Div}( {\varvec{\mathcal {L}}}^{\mathrm{ani}} \nabla d). \end{aligned}$$

What remains now is to superpose the two distinct failure processes emanating from (29), which leads to the rate-independent evolution equation of the phase-field, i.e.,

30 $$\begin{aligned} \displaystyle (1-d){\mathcal {H}}= d - \frac{1}{2} \text {Div} ({\varvec{\mathcal {L}}}\nabla d) , \end{aligned}$$

along with the specific form of the dimensionless crack driving source term

31 $$\begin{aligned} \displaystyle {\mathcal {H}}(t)= \max _{s\in [0,t]}\left[ \langle {\overline{{\mathcal {H}}}}(s) - 1\rangle \right] \quad \text {where} \quad {{\overline{{\mathcal {H}}}}} ={\overline{{\mathcal {H}}}}^{\mathrm{iso}} + {{\overline{{\mathcal {H}}}}}^{\mathrm{ani}}. \end{aligned}$$

Relation (31) yields an irreversible and positive crack driving source term such that the maximum positive value of ${\overline{{\mathcal {H}}}}(s) - 1$ is taken into account in the entire deformation history $s\in [0,t]$, and the Macaulay brackets filter out the positive values for ${{\overline{{\mathcal {H}}}}}(s) -1$ keeping the solid intact until the failure surface is reached. Next, we specify the rate-dependent case in view of (30), i.e.,

32 $$\begin{aligned} \displaystyle \underbrace{\eta {\dot{d}}}_{\text {crack} \,\, \text {evolution}}= \underbrace{(1-d){\mathcal {H}}}_{\text {driving} \,\, \text {force}} - \underbrace{\left[ d - \frac{1}{2} \mathrm{Div}( {\varvec{\mathcal {L}}}\nabla d)\right] }_{\text {geometric} \,\, \text {resistance}}, \end{aligned}$$

where the evolution of the crack is characterized by the balance between the crack driving force and the geometric resistance to the crack (Miehe et al. 2015b).

### A note on the weak formulation and numerical implementation

We now give a brief account to the staggered solution procedure of the multi-field problem associated with the primary field variables ${\varvec{\varphi }}(\mathbf{X},t)$ and $d(\mathbf{X},t)$. An identical temporal as well as spatial discretization scheme is employed for the mechanical and the phase-field problem so as to transform the continuous integral equations into sets of discrete algebraic equations. This set of algebraic equations is solved by a one-pass operator-splitting algorithm in a Newton-type iterative solver for the nodal degrees of freedom.

#### Temporal discretization

The field variables are considered at discrete times $0,t_1,t_2,\ldots ,t_n,t_{n+1},\ldots ,T$ in the process interval [0, *T*]. Focusing on a typical time increment $\tau = t_{n+1}-t_n$ in a solution process, where $t_{n+1}$ and $t_n$ stand for the current and previous time steps, respectively, all field variables at time $t_n$ are assumed to be known, e.g., ${\varvec{\varphi }}_n$ and $d_n$, together with the crack driving source term ${\mathcal {H}}_n$, which is stored as a history variable. In the sequel, the unknown field variables at time $t_{n+1}$ are sought. Note that for the sake of simplicity, all field variables without a subscript such as ${\varvec{\varphi }}$ and *d* are hereinafter evaluated at time $t_{n+1}$.

#### One-pass operator-splitting algorithm

The mechanical and crack phase-field sub-problems can be decoupled by means of a one-pass operator-splitting algorithm, i.e.,

33 $$\begin{aligned} \displaystyle \mathtt{ALGO_{\mathrm{CM}} = ALGO_{\mathrm{C}} \circ ALGO_{\mathrm{M}}}, \end{aligned}$$

for the time increment $\tau$. Such an algorithm is extremely robust, although it slightly underestimates the speed of the crack evolution, see Miehe et al. (2010a) and Gültekin and Holzapfel (2018) for details.

#### Spatial discretization of the mechanical problem

The following notation follows that of Miehe et al. (2010b, 2010a) in which a finite element discretization ${{\mathfrak {T}}}^h$ of the solid ${\mathcal {B}}$ is considered, where *h* denotes the mesh size composed of $E^h$ finite element domains ${\mathcal {B}}^h_e \in {{\mathfrak {T}}}^h$ and $N^h$ global nodal points. In accordance with the discretization ${\mathfrak {T}}^h$, the finite element interpolations of the deformation map and the deformation gradient can be expressed by the state vector

34 $$\begin{aligned} \displaystyle {\mathfrak {c}}^h_\varphi := \{ {\varvec{\varphi }}, \nabla {\varvec{\varphi }}\}^h = \mathbf{B}_\varphi (\mathbf{X})\mathbf{d}_\varphi (t) \end{aligned}$$

in relation to the nodal position vector $\mathbf{d}_\varphi \in {\mathbb {R}}^\delta$, where $\delta \in \{1,2,3\}$ indicates the space dimension, while $\mathbf{B}_\varphi$ serves as a symbolic representation of the global interpolation matrix comprising the shape functions and its derivatives.^1^ For a known phase-field *d* at $t_{n+1}$, the algorithmic potential energy functional related to (25)$_1$ is

35 $$\begin{aligned} \displaystyle \pi _\varphi ^\tau ({\mathfrak {c}}^h_\varphi ) = \int \limits _{{\mathcal {B}}} \left[ \Psi (\nabla {\varvec{\varphi }};d) - \rho _0 {\tilde{{\varvec{\gamma }}}} \cdot {\varvec{\varphi }}\right] \mathrm {d}V - \int \limits _{\partial {\mathcal {S}}_t}{\tilde{\mathbf{t}}}\cdot {\varvec{\varphi }}\mathrm{d}a, \end{aligned}$$

for which the algorithmic form of the variational principle is described as

36 $$\begin{aligned} \displaystyle \mathbf{d}_\varphi = \text{ Arg } \begin{Bmatrix} \displaystyle \underset{{{\mathbf{d}}}_\varphi }{\text{ inf }}\int \limits _{{\mathcal {B}}^h} \pi _\varphi ^\tau ({\mathfrak {c}}^h_\varphi ) \, \mathrm{d}V \end{Bmatrix}. \end{aligned}$$

The respective Euler equation features nonlinear elasticity at finite strains, which is solved by a Newton-type iteration based on a sequence of updates

37 $$\begin{aligned} \displaystyle \mathbf{d}_\varphi&\Leftarrow \mathbf{d}_\varphi - \left( \,\, \int \limits _{{\mathcal {B}}^h} \mathbf{B}_\varphi ^{\mathrm{T}} [ \partial ^2_{{\mathfrak {c}}^h_\varphi \, {\mathfrak {c}}^h_\varphi } \pi _\varphi ^\tau ({\mathfrak {c}}^h_\varphi ) ] \mathbf{B}_\varphi \, \mathrm{d}V \,\, \right) ^{-1} \nonumber \\&\int \limits _{{\mathcal {B}}^h} \mathbf{B}_\varphi ^{\mathrm{T}} [ \partial _{{\mathfrak {c}}^h_\varphi } \pi _\varphi ^\tau ({\mathfrak {c}}^h_\varphi ) ]\, \mathrm{d}V. \end{aligned}$$

#### Spatial discretization of the phase-field problem

In an analogous way to that in Sect. 2.8.3, we write the finite element interpolations of the phase-field and its gradient by

38 $$\begin{aligned} \displaystyle {\mathfrak {c}}^h_d := \{ d, \nabla d \}^h = \mathbf{B}_d(\mathbf{X})\mathbf{d}_d(t), \end{aligned}$$

in relation to the nodal phase-field vector $\mathbf{d}_d \in {\mathbb {R}}^\delta$, where $\delta \in \{1,2,3\}$, while $\mathbf{B}_d$ serves as a symbolic representation of the global interpolation matrix comprising the shape functions and their derivatives. For a known ${\varvec{\varphi }}$ at time $t_{n+1}$, in view of (25)$_2$ the algorithmic potential energy density functional states

39 $$\begin{aligned} \begin{aligned}\pi _d^\tau ({\mathfrak {c}}^h_d) = \int \limits _{{\mathcal {B}}} \left[ \eta \frac{(d - d_n)^2}{2\tau } + g_c\gamma (d,\nabla d; {\varvec{\mathcal {L}}}) - g(d)\Psi _0(\nabla {\varvec{\varphi }}) \right] \mathrm{d}V. \end{aligned} \end{aligned}$$

Then, the discretized form of the variational principle is written as

40 $$\begin{aligned} \displaystyle \mathbf{d}_d = \text{ Arg } \begin{Bmatrix} \displaystyle \underset{{{\mathbf{d}}}_d}{\text{ inf }}\int \limits _{{\mathcal {B}}^h} \pi _d^\tau ({\mathfrak {c}}^h_d) \, \mathrm{d}V \end{Bmatrix}. \end{aligned}$$

The respective Euler equation is linear and can therefore be solved in closed form:

41 $$\begin{aligned} \displaystyle \mathbf{d}_d= & {} \left( \,\, \int \limits _{{\mathcal {B}}^h} \mathbf{B}_d^{\mathrm{T}} [\partial ^2_{{\mathfrak {c}}^h_d \, {\mathfrak {c}}^h_d} \pi _d^\tau ({\mathfrak {c}}^h_d) ] \mathbf{B}_d \, \mathrm{d}V \right) ^{-1} \nonumber \\&\int \limits _{{\mathcal {B}}^h} \mathbf{B}_d^{\mathrm{T}} [\partial _{{\mathfrak {c}}^h_d} \pi _d^\tau ({\mathfrak {c}}^h_d) ]\, \mathrm{d}V . \end{aligned}$$

## Representative numerical examples

Beginning with the material parameter identification for the elastic response, the anisotropic features of the phase-field model are first examined by means of sensitivity analyses, which is followed by the simulation of a nascent aortic dissection within a multi-layered thoracic aortic wall.

### Identification of material parameters

We first fit the elastic constitutive response to experimental data obtained using uniaxial and in-plane simple shear tests performed on medial strips, which are cut from aneurysmatic human thoracic aortas (Sommer et al. 2016). In particular, specimens subjected to uniaxial extension are tested in the circumferential $\theta$- and longitudinal *z*-directions, referred to as ($\theta \theta$) and (*zz*) modes, while in-plane simple shear tests are carried out on the radial *r* plane along the $\theta$- and *z*-directions, indicated by ($r \theta$) and (*rz*) modes, respectively.

The elastic parameters are estimated through a nonlinear least-squares analysis, which is based on a single-objective function $\chi ^2(\mathfrak {p})$ characterizing the sum of squares of the analytical model predictions of the Cauchy stresses $\sigma ^n_{(ij)}$ minus the experimentally determined values ${\bar{\sigma }}^{n}_{(ij)}$, i.e.,

42 $$\begin{aligned} \displaystyle \min _{\mathfrak {p}}\chi ^2(\mathfrak {p}) = \sum _{(ij)\in \mathfrak {m}} \sum _{n=1}^{N_{\mathrm{exp}}^{ (ij)}} ( \sigma _{(ij)}^n - {\bar{\sigma }}_{(ij)}^n )^2. \end{aligned}$$

**Fig. 5:**
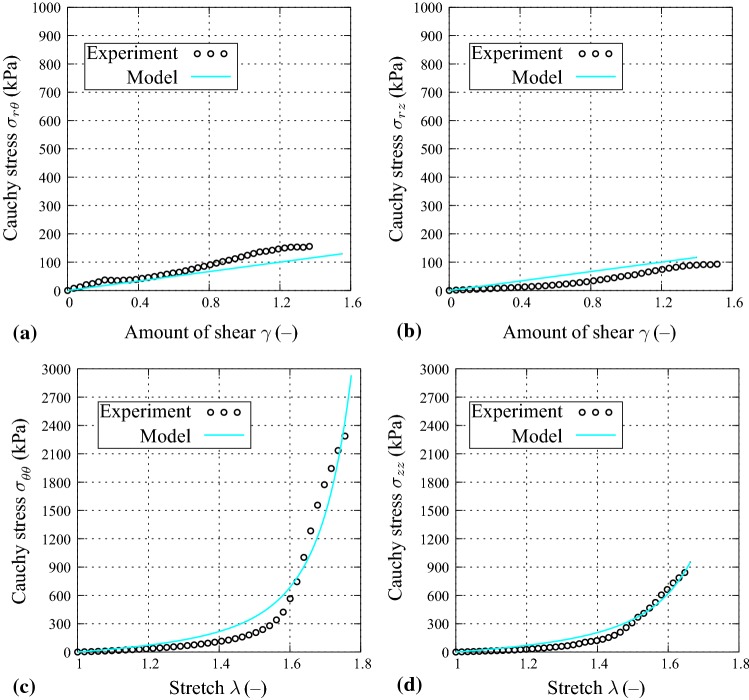
In-plane simple shear and uniaxial extension test data (empty circles) and corresponding model fits (solid curves): Cauchy stress $\sigma$ vs amount of shear $\gamma$ and stretch $\lambda$ for **a**$(r\theta )$; **b** (*rz*); **c**$(\theta \theta )$; **d** (*zz*) modes

The objective function $\chi ^2(\mathfrak {p})$ is minimized with respect to the set of the fitting parameters $\mathfrak {p} = \{ \mu , k_1, k_2, \alpha \}$ of the constitutive model as stated by Holzapfel et al. (2000). Note that $\alpha$ represents the angle between the mean fiber direction and the circumferential $\theta$-direction. The angle $\alpha$ is here considered as a fitting parameter. Furthermore, $\mathfrak {m} = \{ (\theta \theta ), (zz), (r\theta ), (rz)\}$ denotes the set of the aforementioned modes describing the test (*ij*) along with the associated number of data points $N_{\mathrm{exp}}^{ (ij)}$. A MATLAB^®^^2016^ built-in function referred to as *lsqnonlin* is implemented in order to compute the minimization problem. The set of elastic parameters identified is summarized in Table 1 together with the correlation coefficients $R^2_{(ij)}$ and the root-mean-square error $\epsilon$ according to

43 $$\begin{aligned} \displaystyle \epsilon = \displaystyle \frac{\displaystyle \sqrt{\displaystyle \frac{\displaystyle \chi ^2(\mathfrak {p})}{ \displaystyle \sum \nolimits _{(ij)\in \mathfrak {m}} N_{\mathrm{exp}}^{ (ij)}-q}}}{ \displaystyle \sum \nolimits _{(ij)\in \mathfrak {m}} {\bar{\sigma }}^\mathrm{mean}_{(ij)}}, \end{aligned}$$

which is used as a measure for the ‘goodness of fit’ (Holzapfel et al. 2005). Therein, *q* specifies the number of fitting parameters $\mathfrak {p}$, whereas ${\bar{\sigma }}^\mathrm{mean}_{(ij)}$ is the arithmetic mean of the corresponding Cauchy stresses for each mode. The associated hyperelastic constitutive responses are depicted with a reference to experimental data in Fig. 5, which shows favorable agreement.

**Table 1:** Parameters $(\mu , k_1, k_2, \alpha )$ fitted through a nonlinear least-squares analysis in regard to the combined in-plane shear and uniaxial extension tests along with the correlation coefficient $R^2_{(ij)}$ and the corresponding root-mean-square error $\epsilon$

	$\mu \, \hbox {(kPa)}$	$k_1\, \hbox {(kPa)}$	$k_2$ (–)	$\alpha \,(^\circ )$
Parameter	83.509	101.651	4.173	44.705
Correlation coefficient	$R^2_{(r\theta )} = 0.991$	$R^2_{(rz)}=0.978$	$R^2_{(\theta \theta )} = 0.979$	$R^2_{(rz)}=0.990$
Root-mean-square error		$\epsilon = 0.104$		

### Sensitivity analysis of the anisotropy parameter $\omega _{\mathrm{M}}$

**Fig. 6:**
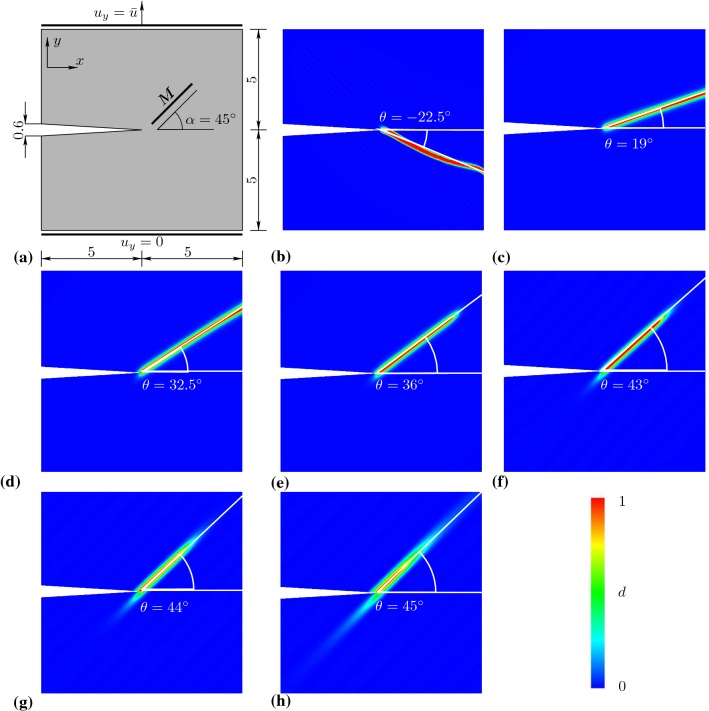
**a** Geometry of a squared single-edge notched domain assumed with plane strain conditions is extended in the *y*-direction (all dimensions are in millimeters). Diffusive crack patterns with distinct crack angles $\theta$ in relation to different $\omega _{\mathrm{M}}$ are visualized for **b**$\omega _{\mathrm{M}} \approx -1$; **c**$\omega _{\mathrm{M}}=1$; **d**$\omega _{\mathrm{M}}=5$; **e**$\omega _{\mathrm{M}}=10$; **f**$\omega _{\mathrm{M}}=50$; **g**$\omega _{\mathrm{M}}=100$; **h**$\omega _{\mathrm{M}}=500$

To demonstrate how sensitive the crack path is with respect to the anisotropy parameter $\omega _{\mathrm{M}}$, a plane strain boundary-value problem is analyzed. In particular, a squared single-edge notched domain is considered with 38,800 quadrilateral elements, which are connected by 39,295 nodes. The material exhibits anisotropy characterized by a single family of fibers with mean orientation $\mathbf{M}$, orientated at an angle $\alpha =45^\circ$ with respect to the *x*-axis, see Fig. 6a. While the bottom edge of the domain is fixed in the *y*-direction ($u_y = 0$), the top edge is subjected to a monotonically increasing vertical displacement ($u_y = {\bar{u}}$).

The material parameters used for the simulations are $\mu = 1.0\,\hbox {kPa}$, $k_1 = 1.0\,\hbox {kPa}$, $k_2 = 1.0$ together with the bulk modulus $\kappa = 3.0\,\hbox {kPa}$. The phase-field parameters are chosen as $g_{\mathrm{c}}^{\mathrm{iso}}/l = 10^{-2}\,\hbox {kPa}$ and $g_{\mathrm{c}}^{\mathrm{ani}}/l = 10^{-2}\,\hbox {kPa}$, with the length scale $l = 0.1\,\hbox {mm}$ satisfying $l>2h$ in order to resolve the diffusive crack surface, where *h* refers to the minimum mesh size.

Figure 6b–h depicts the influence of the anisotropy parameter $\omega _{\mathrm{M}}$ on the crack pattern, starting with $\omega _{\mathrm{M}} \approx -1$ up to $\omega _{\mathrm{M}}=500$, where the crack starts to follow the orientation of fibers ($\alpha =45^\circ$), as $\omega _{\mathrm{M}}$ increases, which complies with the findings of Teichtmeister et al. (2017) in the small strain context. In fact, the crack path becomes almost parallel to the fibers for $\omega _{\mathrm{M}} = 500$. To elaborate on the results obtained, let us substitute (8) into (7)$_2$ which reshapes the anisotropic crack surface density to

44 $$\begin{aligned} \displaystyle \gamma (d,\nabla d; {\varvec{\mathcal {L}}})= \ & {} \frac{d^2}{2l} + \frac{l}{2} \nabla d \cdot \nabla d \nonumber \\&+ \frac{l}{2} \omega _{\mathrm{M}} (\nabla d \cdot \mathbf{M}) (\mathbf{M}\cdot \nabla d). \end{aligned}$$

From (44) it is easily discernible that $\omega _{\mathrm{M}}$ serves as a penalty parameter, which enforces $\angle (\nabla d;\,\mathbf{M}) = 0^{\circ }$ as $\omega _{\mathrm{M}} \rightarrow \infty$, making the crack path aligned with the mean orientation $\mathbf{M}$ of the fiber family. It is worth emphasizing that a slight crack kinking is observed for $\omega _{\mathrm{M}} \approx -1$ which may be a result of the limit imposed by the ellipticity condition and possible multiple minima of the energy encountered on the path. The anisotropy parameter $\omega _{\mathrm{M}}$ is responsible for the transition of the fracture mechanism from fiber bridging to matrix cracking as its value increases, see Fig. 1b.

Stability becomes an issue for cases where $\omega _{\mathrm{M}} \ge 10$, which causes a termination of the computation prematurely for the standard displacement-based *Q1* finite element formulation. Such a predicament is not observed within the small strain context (Li et al. 2015; Teichtmeister et al. 2017). The reason may be found in both the finite strain framework employed here, and the exponential stiffening attributed to the fibers that might have hampered the model stability for $d \rightarrow 1$.

A theoretical prediction for the crack angle $\theta$ is suggested by Takei et al. (2013). These authors translate the maximum energy release rate concept, see Erdoğan and Sih (1963), into a graphical representation by analogy with the so-called Wulff’s plot for crystal growth (Herring 1951). The graphical construction consists of a polar plot of the inverse anisotropic critical energy release rate $G_\mathrm{c}^{-1}(\alpha ,\theta )$ and a line plot of the anisotropic energy release rate $G(\theta )$ imposed by loading, which is tangent to the polar plot. This tangency marks the angle with which the crack propagates in the anisotropic continuum. Although this method is applicable within the small strain context, its use for finite deformations is debatable, as the experimental analysis in Takei et al. (2013) neglects elastic stretching.

**Fig. 7:**
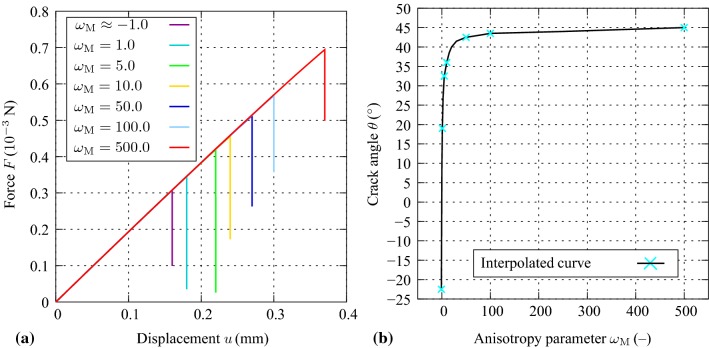
**a** Force-displacement curve with respect to different anisotropy parameters $\omega _{\mathrm{M}}$; **b** sensitivity of the crack angle $\theta$ in relation to the anisotropy parameter $\omega _{\mathrm{M}}$

Figure 7a, b shows the corresponding force–displacement curves along with the sensitivity of the crack angle $\theta$ with respect to the anisotropy parameter $\omega _{\mathrm{M}}$. The force required for fracture is remarkably elevated by the increase of $\omega _{\mathrm{M}}$, which can be attributed to the increased effective length scale parameter due to $\omega _{\mathrm{M}}$ (Gültekin et al. 2016), thereby resulting in a greater geometric resistance against fracture. Aside from that, a notably sensitive character of the crack angle $\theta$ associated with relatively low values of $\omega _{\mathrm{M}}$ is followed by a saturation-type behavior for larger values of $\omega _{\mathrm{M}}$ (>100). Note that in order to obtain the traceable curve in Fig. 7b, additional computations with varying $\omega _{\mathrm{M}}$ were performed.

### Aortic dissection propagation

This example marks a proof of concept in regard to the 3-D modeling of an aortic dissection propagation upon its initiation, which delineates a helical pattern within the multi-layered wall structure, specifically inside a medial sub-layer in the neighborhood of the prescribed initial tear due to stress concentrations.

#### Geometry and material

**Fig. 8:**
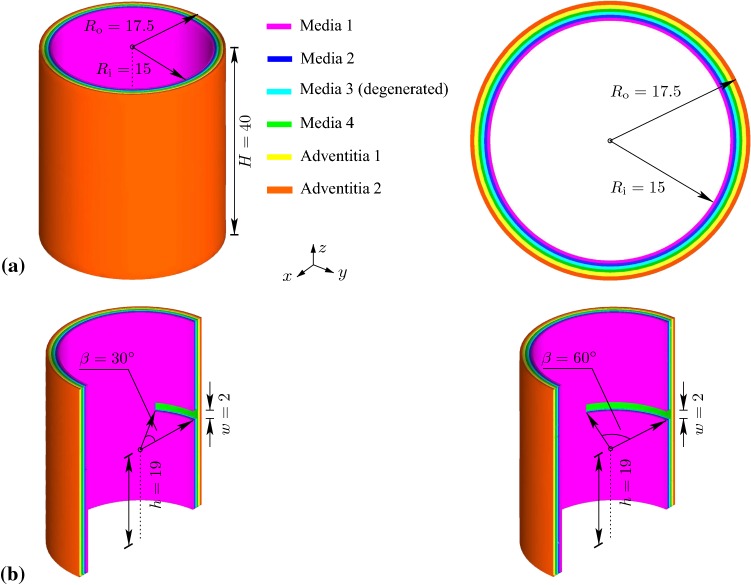
**a** Idealized geometry of an extracted 3-D segment obtained from a human ascending aorta composed of four medial sub-layers associated with the color codes pink, blue, cyan and green, and two adventitial sub-layers represented by yellow and orange color. Note that *media 3* refers to a degenerated layer (with lower strength); **b** sliced view of the geometry depicting the prescribed initial tear size depending on the varying parameter $\beta$ that regulates the length of the tear. All dimensions are in millimeters

A segment with $H = 40\,\hbox {mm}$ length is isolated from the human ascending aorta possessing typical geometrical values, measured at the end-diastolic phase, with inner and outer aortic radii $R_{\mathrm{i}} = 15$ and $R_{\mathrm{o}} = 17.5\,\hbox {mm}$, respectively, as reported by Mao et al. (2008). The geometrical setup is tailored for an idealized geometry, which features a cylindrical tube consisting of 6 layers. Starting from the endothelium, the first four layers (with color codes pink, blue, cyan, green, as illustrated in Fig. 8a) belong to the combination of intima and media, while the outermost two layers represent the adventitia (with color codes yellow and orange). Each of the medial and adventitial sub-layers has reference thicknesses of $T_{\mathrm{med}} = 0.375$ and $T_{\mathrm{adv}} = 0.5\,\hbox {mm}$, respectively.

The initial tear size and tear-shape are assumed to be *a priori* known and span three medial sub-layers across the thickness of the wall, i.e., from the endothelium up to *media 4* sub-layer. Notches with varying length $\pi R_\mathrm{i}\beta /180^\circ$, where $\beta \in \{30^\circ , 60^\circ \}$, with a width of $w = 2\,\hbox {mm}$, are incorporated into the solid model to examine the influence of the initial tear size on the progression of the dissection, as depicted in Fig. 8b.

**Table 2:** Elastic and crack phase-field parameters related to the individual layers used for the extension–inflation–torsion analysis

Layer	$\mu \, \hbox {(kPa)}$	$k_1\, \hbox {(kPa)}$	$k_2$ (–)	$\alpha \, (^\circ )$	$g_{\mathrm{c}}^{\mathrm{iso}}/l\, \hbox {(kPa)}$	$g_{\mathrm{c}}^{\mathrm{ani}}/l\, \hbox {(kPa)}$	$\omega _{\mathrm{M}}$ (–)
Healthy media	100.21	121.98	5.01	44.71	100.0	300.0	$10^3$
Degenerated media	83.51	101.65	4.17	44.71	6.0	18.0	$10^3$
Adventitia	200.0	400.0	4.0	44.71	100.0	300.0	$10^3$

The parameters identified in Sect. 3.1 stand for a degenerated media whose constitutive response exhibits a mechanical degradation which is expressed by the related material parameters. In particular, the degenerated media corresponds to the sub-layer *media 3*. Since respective tests on the healthy medial specimens from the same sample are lacking, we have increased the values of the constitutive parameters $\mu$, $k_1$, $k_2$ by 20% and attributed them to *media 1*, *media 2* and *media 4*, as summarized in Table 2. Because of the lack of experimental data of the adventitial layer, we assume a relatively stiffer response of the healthy adventitial layer with respect to the healthy medial layer, and use (rather) arbitrary constitutive parameters.

As for the phase-field parameters, a direct measurement is generally impeded by the size effect during a rupture test. Most of the extant studies solely report the ultimate stress and stretch values that evoke a rather rudimentary information on the tissue strength. Therefore, arbitrary values for $g_{\mathrm{c}}^i/l$ are considered for the sake of proving the concept elucidated in the present study. Nonetheless, the anisotropy parameters are specified in the light of the sensitivity analysis, as described in Sect. 3.2.

#### Mesh and fiber orientation

**Fig. 9:**
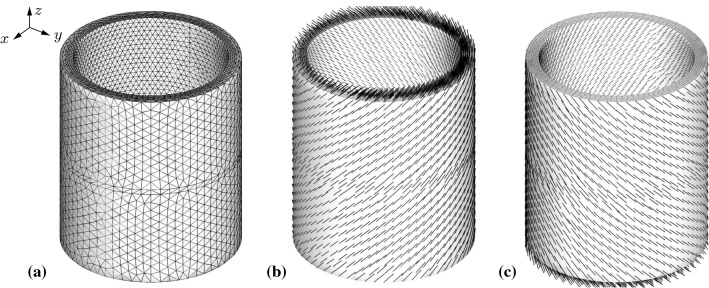
**a** Finite element mesh of the idealized cylindrical tube with an initial tear; **b** orientation of first the family of fibers (denoted by $\mathbf{M}$); **c** second family of fibers (denoted by $\mathbf{M}^\prime$)

The corresponding finite element meshes consist of four-node tetrahedral elements, see Table 3, with a constant length scale parameter $l = 0.1875\,\hbox {mm}$. Figure 9a depicts a typical meshed geometry for the problem considered. In addition, for the sake of simplicity, the fitted angle $\alpha = 44.71$ (see Table 1) between the orientation of the fibers $\{\mathbf{M}, \mathbf{M}^\prime \}$ and the circumferential direction for *media 3* is applied to each of the sub-layers of media and adventitia in a discrete sense, as visualized in Fig. 9b, c, respectively.

**Table 3:** Total number of nodes and elements pertaining to each geometry given in Fig. 8b designed according to the parameters controlling the length ($\beta$) and width (*w*) of the initial tear

Geometry	# of nodes	# of elements
$\beta =30^\circ ,\,w=2$	20,109	101,400
$\beta =60^\circ ,\, w=2$	20,943	105,571

**Fig. 10:**
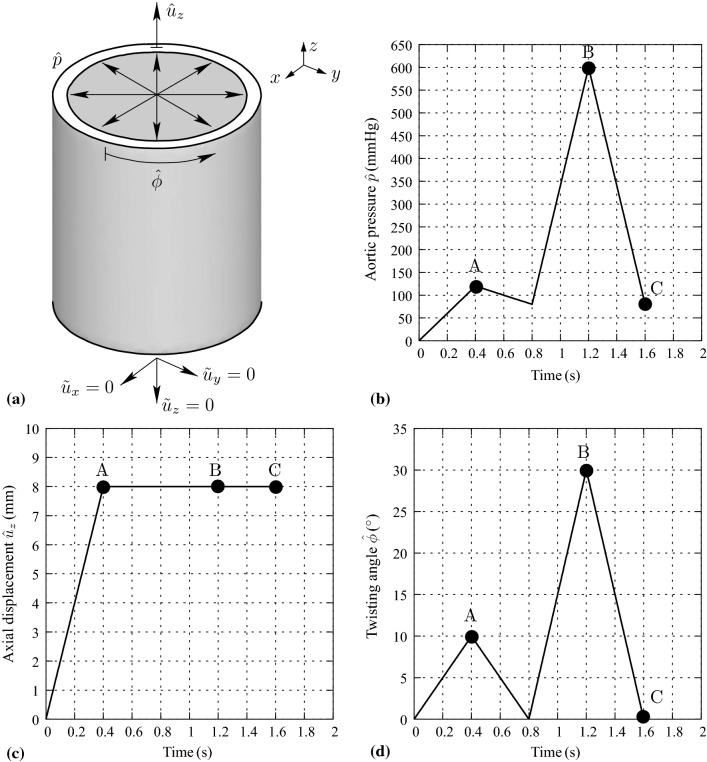
**a** Displacements are constrained at the bottom plane where $z = 0\,\hbox {mm}$, along the *x*-, *y*-, and *z*-directions, required to twist the specimen at the top plane at $z = 40\,\hbox {mm}$; loading conditions for the extension–inflation–torsion test are realized by one physiological and one supra-physiological cycle in a saw-tooth manner with regard to **b** aortic pressure ${\hat{p}}$; **c** axial displacement ${\hat{u}}_z$ (remains constant after the peak in the physiological cycle is reached); **d** twisting angle ${\hat{\phi }}$. Snapshots of the results are shown at instants $\mathrm{A}$, $\mathrm{B}$, and $\mathrm{C}$ at time $t \in \{0.4, 1.2, 1.6 \}$ corresponding to the peak physiological, supra-physiological loading states, and to the end of the simulation

#### Boundary and loading conditions

The solid domain is subjected to an extension–inflation–torsion test with appropriate boundary conditions, see Fig. 10a, which is performed in two loading cycles. The first loading cycle refers to a physiological state, while the second cycle refers to a (rather assumed) supra-physiological loading state. In particular, the physiological aortic pressure ${\hat{p}}$ ranges between 80 and 120 mmHg, while the supra-physiological state reaches an assumed peak pressure value of 600 mmHg reproducing hypertension, the most common predisposing factor of patients suffering aortic dissection (Mussa et al. 2016). Both of them are applied on the inner surface of the wall in a saw-tooth loading manner, as depicted in Fig. 10b. Arteries are significantly pre-stretched, and the axial deformations are close to zero during pressure cycles (Schulze-Bauer et al. 2003). Following the study by Horný et al. (2014) on the age-dependence of the axial pre-stretch values of human abdominal aortas, a representative axial displacement of ${\hat{u}}_z = 8\,\hbox {mm}$, which is the equivalent of an axial stretch of $\lambda _z = 1.2$, is applied during the physiological state and maintained during the supra-physiological loading cycle, see Fig. 10c. Experimental measurements suggest an end-systolic twisting angle ${\hat{\phi }}$ for a healthy left ventricle which ranges between 8 and $12^\circ$ in the physiological state, as reported by Carreras et al. (2011). These values can also be predicted for the ascending part of the aorta. Nonetheless, a higher value of the twisting angle may occur. We assume here a peak twist angle of $10^\circ$ and $30^\circ$ with regard to the physiological and supra-physiological scenarios, as illustrated in Fig. 10d.

**Fig. 11:**
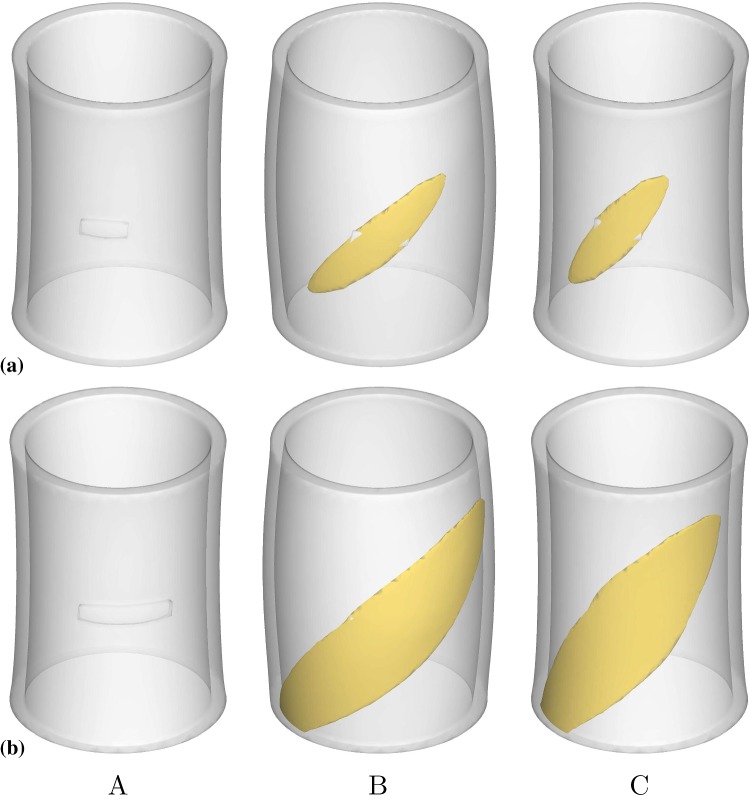
3-D evolution of the crack phase-field in the degenerated *media 3* at instants $\mathrm{A}$, $\mathrm{B}$ and $\mathrm{C}$, according to Fig. 10, in regard to varying tear sizes: **a**$\beta = 30^\circ$, $w = 2$; **b**$\beta = 60^\circ$, $w = 2$ (see Fig. 8b). Isosurfaces are used to visualize the damage zone corresponding to $d \ge 0.8$

#### Simulations and numerical results

**Fig. 12:**
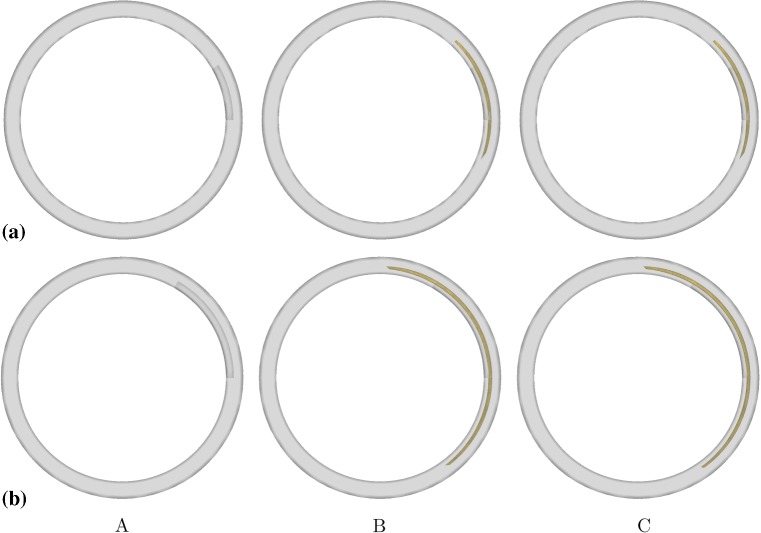
Circumferential evolution of the crack phase-field in the degenerated *media 3* at instants $\mathrm{A}$, $\mathrm{B}$ and $\mathrm{C}$, according to Fig. 10, when viewed from the top plane at $z = 40\,\hbox {mm}$ in regard to varying tear sizes: **a**$\beta = 30^\circ$, $w = 2$; **b**$\beta = 60^\circ$, $w = 2$ (see Fig. 8b). Isosurfaces are used for the cross-sectional view to visualize the damage zone corresponding to $d \ge 0.8$

In view of the loading scenario described in Sect. 3.3.3, all simulations start with a time increment of $\tau = 10^{-2}$, which is decreased up to $\tau = 10^{-4}$ when a stability issue occurs. The 3-D evolution of the crack phase-field *d* in the degenerated medial sub-layer (*media 3*) at instants $\mathrm{A}$, $\mathrm{B}$, and $\mathrm{C}$ is depicted in Fig. 11. In particular the damage zone of the thoracic aortic segment is shown, where $d \ge 0.8$ for varying initial tear sizes, as specified in Fig. 8b. As a matter of fact, none of the geometric descriptions result in an acute/excessive damage zone around the tear at instant $\mathrm{A}$ referring to the peak physiological loading state indicated in Fig. 10. The cross-sectional view obtained from the top plane at $z = 40\,\hbox {mm}$ in Fig. 12 also illustrates the situation at instant $\mathrm{A}$.

**Fig. 13:**
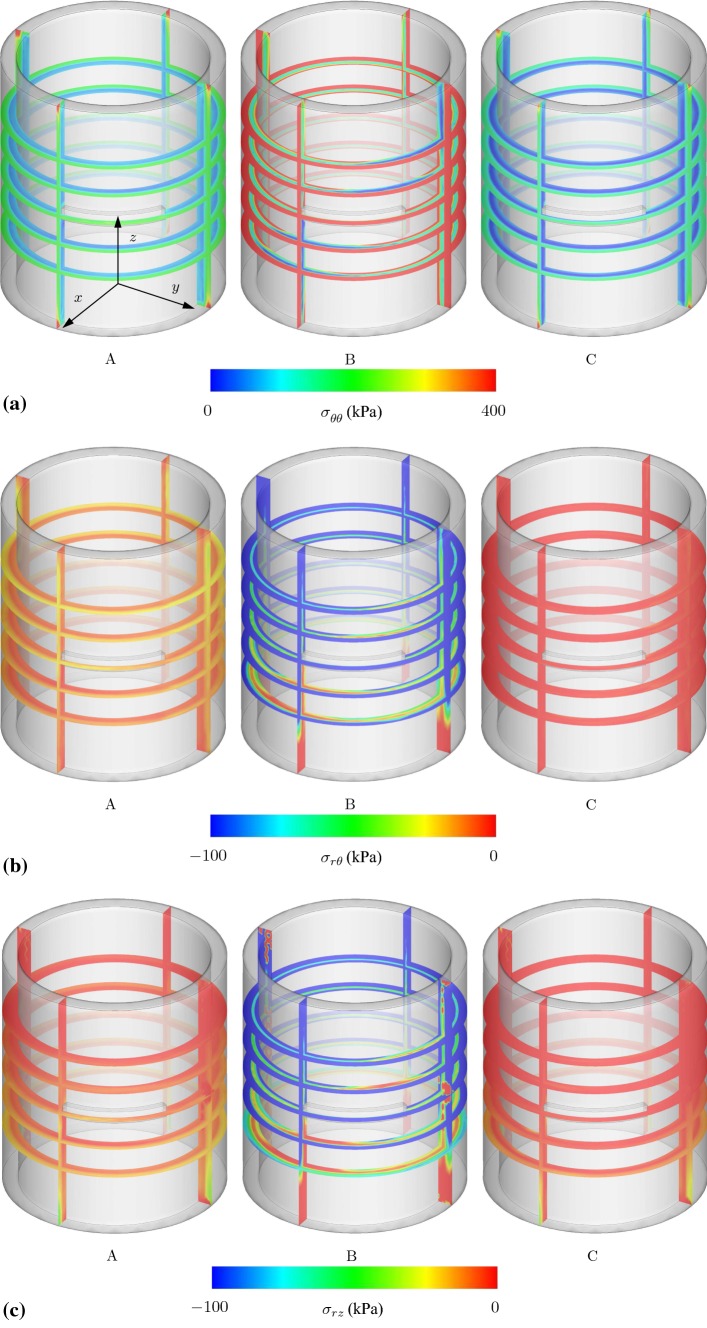
Distributions of **a** circumferential Cauchy stress $\sigma _{\theta \theta }$, **b** in-plane shear stress $\sigma _{r \theta }$ and **c** in-plane shear stress $\sigma _{r z}$ with the initial tear size $\beta = 60^\circ$, $w=2$ (see Fig. 8b) obtained at the instants $\mathrm{A}$, $\mathrm{B}$ and $\mathrm{C}$. Transparency is used with slices extracted on planes at $z=10$, 15, 20, 25, 30, then at $x=0$, and $y=0$ (for the related coordinate system see **a**)

The analysis shows a crack initiation around the initial tear due to stress concentration, which propagates in a specific manner by aligning with the direction of the first fiber family, as seen in Fig. 11, where $d \ge 0.8$; the crack follows a rather helical path in the 3-D domain. A special focus is now given to the related stress distributions in Fig. 13a–c (for the case of Fig. 11b). These figures indicate a significant loss of the load-bearing capacity of the degenerated medial sub-layer (*media 3*) at instant $\mathrm{B}$ within the damage zone, compare with Fig. 11b. Such a mechanical degradation is undoubtedly accompanied by a loss of intactness in the respective sub-layer. In reality it causes the blood to enter the wall through the initial tear which will peel off *media 1* and *media 2* from *media 4*, *adventitia 1* and *adventitia 2*. A cascade of supra-physiological cycles would trigger even more tearing as the blood jets through the medial sub-layer yielding a false lumen next to the true one. It is also worth highlighting that larger initial tears provide larger damage zones, which are associated with higher stress concentrations, as demonstrated in Fig. 11a, b. For the sake of completeness, the end-simulation snapshots for the phase-field ($d \ge 0.8$) are shown at instant $\mathrm{C}$ in Figs. 11, 12 and 13. The results are in agreement with the model consideration that features an irreversible character.

## Discussion

In the light of the mechanical tests documented by Sommer et al. (2016) and Haslach et al. (2018), the focus is placed on the ubiquitous (elastic) mechanical factors involved in aortic dissection, particularly on the normal and in-plane shear stresses. The elastic material properties are identified from experimental data, as depicted in Fig. 5. However, it is most likely the case that a certain amount of damage, e.g., stress softening, is induced prior to the ultimate stresses, which motivates further studies on the parameter quantification. Another important observation we identified during the analyses is that displacement driven tests such as uniaxial extension, shear and peel tests seem to overestimate the rupture properties. In particular, for a dissecting aortic tissue, basically no severely damaged zone is achieved when the energy release rates are used from the literature (Sommer et al. 2008, 2016; Tong et al. 2014; Leng et al. 2018).

Separation of the medial lamellae seems to follow a twofold mechanism. Inhomogeneity in the respective mechanical properties results in in-plane circumferential and longitudinal shear components ($\sigma _{r \theta }$, $\sigma _{r z}$) during inflation of the aortic segment. They are probably responsible for the rupture of interfacial bonds between two adjacent lamellae which is in line with the fracture mechanisms described in Sect. 1.1 (fiber pull-out, fiber bridging, fiber debonding, matrix failure). This rupture enables the blood to enter the interface through the initial tear, while the lamellae have mostly lost their mechanical resistance as $d \ge 0.8$. In a sense, the crack propagation seems to follow a *mode II* type of fracture rather than *mode I*. In a nutshell, inhomogeneous in-plane shear deformations catalyzed by the heterogeneous material properties evoke *mode II* fracture in the form of the failure mechanisms shown in Fig. 1b. That forms the basis for the separation of the medial lamellae leading to aortic dissection as observed at the macro-scale.

A systematic characterization of the elastic and rupture properties of the aorta in health and disease is of particular importance to fracture models in order to cope with the elusive phenomenon of aortic rupture. In fact, constituent-specific mechanical tests (uniaxial extension, shear, peel tests etc.) on adjacent tissue strips extracted from the ascending and descending parts of the aorta should be performed after elastase (breaks down elastin) and collagenase (breaks peptide bonds in collagen). Such enzymatic removals (enzymolyses) of elastin and collagen have been studied by, e.g., Roach and Burton (1957) and more recently by Schriefl et al. (2015). To a certain extent this enables a better understanding of the mechanical role of elastin/collagen and would allow a refined quantitative assessment of the individual rupture behavior via the identification of the degradation parameters $a_{\mathrm{iso}}$, $a_{\mathrm{ani}}$ (see Sect. 2.5) and the critical fracture energies $g_{\mathrm{c}}^{\mathrm{iso}}$, $g_\mathrm{c}^{\mathrm{ani}}$ (see Sect. 2.7.2). The determination of the layer-specific elastic and rupture properties of the ascending and descending aortas would definitely enhance our understanding of the role of altered mechanical wall properties, and better inform computational models.

It has been speculated that PGs contribute to the mechanics of arterial walls by linking the individual collagen fibers together. In this respect, matrilysins (Ross and Pawlina 2011) can be used before mechanical tests to better decipher the role of PGs on the mechanical wall response. The reader is also referred to the computational study of Roccabianca et al. (2014), which presents finite element simulations that support a hypothesis for the initiation of local delaminations that subsequently propagate as dissections. In particular, according to the hypothesis of Humphrey (2013), the authors of Roccabianca et al. (2014) show that the pooling of GAGs/PGs in the medial layer of a thoracic aorta can lead to significant stress concentrations through intra-lamellar Donnan swelling pressures that disrupt the normal cell-matrix interactions and delaminate the layered micro-structure of the aortic wall. The pooling of GAGs/PGs may be initiated by an increased transforming growth factor-$\beta$ (TGF-$\beta$). As a consequence, a significant reduction of the radial elastic properties due to elastic fiber breakage may take place between the elastic laminae before the event of an aortic dissection (MacLean et al. 1999). Such a condition can initiate a local delamination and/or altered mechanosensitive cellular response leading to dysregulated wall homeostasis (Humphrey 2013). Consequently, constitutive models need to be improved by including more realistic mechanisms, as described here, and we need to better identify under which load combination the crack is initiated so that clinical events can better be simulated.

It should also be underlined that the simulations we documented in Sect. 3.3 provide limited validity from a quantitative point of view. Nevertheless, to show the capability of the algorithm as a proof of concept to determine the incipient progression of a dissection is the ultimate goal of this example. The phase-field approach can also be combined with XFEM in order to visually capture the delamination phenomenon and the formation of a false lumen. Thereby, the global problem would probably lead to a dynamical problem which might be a computationally troublesome task to undertake via implicit solvers; therefore, an explicit analysis is recommended.

In our previous study we demonstrated the numerical performance of the crack phase-field model by analyzing uniaxial extension and simple shear fracture tests performed on tissue samples obtained from a human aneurysmatic thoracic aorta (Gültekin et al. 2016). We need to collect more experimental data from human tissues harvested from dissected aortas in order to better validate our model via comparison. Future modeling efforts should also be informed by imaging data extracted from cardiac computed tomography or cardiac magnetic resonance angiography (Baliga et al. 2014; Doyle and Norman 2016). The best would be having access to imaging data from the aorta before and after the event of a dissection from the same patient. Such data should also capture the origin of the crack with a resolution allowing detailed 3D reconstruction. Such geometrical data would also better allow to show the potential of the proposed methodology.

## Conclusion

The present contribution addresses the incipient aortic dissection that takes place in a degenerated medial sub-layer in the vicinity of a prescribed initial tear. The aortic wall segment comprises several layers of intima/media and adventitia with various mechanical properties. The proposed crack phase-field model when combined with appropriate hyperelastic constitutive relations captures the anisotropic rupture behavior of the aortic wall. Together with improved imaging techniques and computational power, high-end mechanistic models can further be calibrated and optimized to shed more light on the mechanical aspects of aortic dissections.

## Footnotes

1 *FE discretization:* For a plane strain problem ($\delta = 2$) the state vector of the deformation field reads $[{\mathfrak {c}}^h_\varphi ] = [ \varphi _1, \varphi _2, \varphi _{1,1}, \varphi _{2,2}, \varphi _{1,2}, \varphi _{2,1} ]$ for which the finite element interpolation matrix takes on the form

$$\begin{aligned}{}[\mathbf{B}_\varphi ]^e_I = \begin{bmatrix} N&0&N,_1&0&N,_2&0 \\ 0&N&0&N,_2&0&{N,}_1 \end{bmatrix}^{\mathrm{T}}_I \end{aligned}$$

in terms of the shape function $N_I$ at node *I* and its derivatives. Analogously, the state vector of the phase-field reads $[{\mathfrak {c}}^h_d] = [ d, d,_1, d,_2]$ for which the finite element interpolation matrix gives

$$\begin{aligned}{}[\mathbf{B}_d]^e_I = \begin{bmatrix} N&N,_1&N,_2 \end{bmatrix}^{\mathrm{T}}_I. \end{aligned}$$

## References

[CR2] Ambati M, Gerasimov T, De Lorenzis L (2015). Phase-field modeling of ductile fracture. Comput Mech.

[CR3] Anderson TL (2005). Fracture mechanics: fundamentals and applications.

[CR4] Baliga RR, Nienaber CA, Bossone E, Oh JK, Isselbacher EM, Sechtem U, Fattori R, Raman SV, Eagle KA (2014). The role of imaging in aortic dissection and related syndromes. JACC Cardiovasc Imaging.

[CR5] Borden MJ, Hughes TJR, Landis CM, Anvari A, Lee IJ (2016). A phase-field formulation for fracture in ductile materials: Finite deformation balance law derivation, plastic degradation, and stress triaxiality effects. Comput Meth Appl Mech Eng.

[CR6] Carreras F, Garcia-Barnes J, Gil D, Pujadas S, Li CH, Suarez-Arias R, Leta R, Alomar X, Ballester M, Pons-Llado G (2011). Left ventricular torsion and longitudinal shortening: two fundamental components of myocardial mechanics assessed by tagged cine-MRI in normal subjects. Int J Cardiovasc Imaging.

[CR7] Carson MW, Roach MR (1990). The strength of the aortic media and its role in the propagation of aortic dissection. J Biomech.

[CR8] Cheng Z, Riga C, Chan J, Hamady M, Wood NB, Cheshire NJW, Xu Y, Gibbs RGJ (2013). Initial findings and potential applicability of computational simulation of the aorta in acute type B dissection. J Vasc Surg.

[CR9] Cherry KJ, Dake MD, Hallett JW, Mills JL, Earnshaw J, Reekers JA, Rooke TW (2009). Aortic dissection. Comprehensive vascular and endovascular surgery.

[CR10] Clouse WD, Hallett JW, Schaff HV, Spittell PC, Rowland CM, Ilstrup DM, Melton LJ (2004). Acute aortic dissection: population-based incidence compared with degenerative aortic aneurysm rupture. Mayo Clin Proc.

[CR11] Criado FJ (2011). Aortic dissection: a 250-year perspective. Tex Heart Inst J.

[CR12] Doyle BJ, Norman PE (2016). Computational biomechanics in thoracic aortic dissection: today’s approaches and tomorrow’s opportunities. Ann Biomed Eng.

[CR13] Dunning DW, Kahn JK, Hawkins ET, O’Neill WW (2000). Iatrogenic coronary artery dissections extending into an involving the aortic root. Catheter Cardiovasc Interv.

[CR14] Erdoğan F, Sih GC (1963). On the crack extension in plates under plane loading and transverse shear. ASME J Basic Eng.

[CR15] Ferrara A, Pandolfi A (2010). A numerical study of arterial media dissection processes. Int J Fract.

[CR16] Francfort GA, Marigo J-J (1998). Revisiting brittle fracture as an energy minimization problem. J Mech Phys Solids.

[CR17] Gasser TC, Holzapfel GA (2006). Modeling the propagation of arterial dissection. Eur J Mech A Solids.

[CR18] Gasser TC, Ogden RW, Holzapfel GA (2006). Hyperelastic modelling of arterial layers with distributed collagen fibre orientations. J R Soc Interface.

[CR19] Gültekin O, Holzapfel GA, Oñate O, Peric D, de Souza Neto E, Chiumenti M (2018). A brief review on computational modeling of rupture in soft biological tissues. Computational methods in applied sciences. Advances in computational plasticity. A book in honour of D. Roger J. Owen.

[CR20] Gültekin O, Dal H, Holzapfel GA (2016). A phase-field approach to model fracture of arterial walls: theory and finite element analysis. Comput Methods Appl Mech Eng.

[CR21] Gültekin O, Dal H, Holzapfel GA (2018). Numerical aspects of anisotropic failure in soft biological tissues favor energy-based criteria: a rate-dependent mixed crack phase-field model. Comput Methods Appl Mech Eng.

[CR22] Haslach HW, Siddiqui A, Weerasooriya A, Nguyen R, Roshgadol J, Monforte N, McMahon E (2018). Fracture mechanics of shear crack propagation and dissection in the healthy bovine descending aortic media. Acta Biomater.

[CR23] Herring C (1951). Some theorems on the free energies of crystal surfaces. Phys Rev.

[CR24] Holzapfel GA (2000). Nonlinear solid mechanics. A continuum approach for engineering.

[CR25] Holzapfel GA, Ogden RW (2017). Comparison of two model frameworks for fiber dispersion in the elasticity of soft biological tissues. Eur J Mech A Solids.

[CR26] Holzapfel GA, Ogden RW (2017). On fiber dispersion models: exclusion of compressed fibers and spurious model comparisons. J Elast.

[CR27] Holzapfel GA, Gasser TC, Ogden RW (2000). A new constitutive framework for arterial wall mechanics and a comparative study of material models. J Elast.

[CR28] Holzapfel GA, Sommer G, Gasser CT, Regitnig P (2005). Determination of layer-specific mechanical properties of human coronary arteries with non-atherosclerotic intimal thickening, and related constitutive modeling. Am J Physiol Heart Circ Physiol.

[CR29] Horný L, Netušil M, Voňavková T (2014). Axial prestretch and circumferential distensibility in biomechanics of abdominal aorta. Biomech Model Mechanobiol.

[CR30] Howard DP, Banerjee A, Fairhead JF, Perkins J, Silver LE, Rothwell PM, Oxford Vascular Study (2013) Population-based study of incidence and outcome of acute aortic dissection and premorbid risk factor control: 10-year results from the Oxford mboxVascular mboxStudy. Circulation 127:2031–2037 10.1161/CIRCULATIONAHA.112.000483 PMC6016737 23599348

[CR31] Humphrey JD (2013). Possible mechanical roles of glycosaminoglycans in thoracic aortic dissection and associations with dysregulated transforming growth factor-$$\beta $$. J Vasc Res.

[CR32] Khan IA, Nair CK (2002). Clinical, diagnostic, and management perspectives of aortic dissection. Chest.

[CR33] Leng X, Zhou B, Deng X, Davis L, Lessner SM, Sutton MA, Shazly T (2018). Experimental and numerical studies of two arterial wall delamination modes. J Mech Behav Biomed Mater.

[CR34] Li B, Peco C, Millán D, Arias I, Arroyo M (2015). Phase-field modeling and simulation of fracture in brittle materials with strongly anisotropic surface energy. Int J Numer Methods Eng.

[CR35] MacLean NF, Dudek NL, Roach MR (1999). The role of radial elastic properties in the development of aortic dissections. J Vasc Surg.

[CR36] Malvindi PG, Pasta S, Raffa GM, Livesey S (2017). Computational fluid dynamics of the ascending aorta before the onset of type A aortic dissection. Eur J Cardio Thorac.

[CR37] Mao SS, Ahmadi N, Shah B, Beckmann D, Chen A, Ngo L, Flores FR, Gao YL, Budoff MJ (2008). Normal thoracic aorta diameter on cardiac computed tomography in helathy asymptomatic adult: impact of age and gender. Acad Radiol.

[CR1] MATLAB Release R2016a. The MathWorks Inc. (2016), Natick, MA, USA

[CR38] Miehe C (2011). A multi-field incremental variational framework for gradient-extended standard dissipative solids. J Mech Phys Solids.

[CR39] Miehe C, Hofacker M, Welschinger F (2010). A phase field model for rate-independent crack propagation: robust algorithmic implementation based on operator splits. Comput Methods Appl Mech Eng.

[CR40] Miehe C, Welschinger F, Hofacker M (2010). Thermodynamically consistent phase-field models of fracture: variational principles and multi-field FE implementations. Int J Numer Methods Eng.

[CR41] Miehe C, Hofacker M, Schänzel L-M, Aldakheel F (2015). Phase field modeling of fracture in multi-physics problems. Part II. Coupled brittle-to-ductile failure criteria and crack propagation in thermo-elastic–plastic solids. Comput Methods Appl Mech Eng.

[CR42] Miehe C, Schänzel L-M, Ulmer H (2015). Phase field modeling of fracture in multi-physics problems. Part I. Balance of crack surface and failure criteria for brittle crack propagation in thermo-elastic solids. Comput Methods Appl Mech Eng.

[CR43] Miehe C, Dal H, Schänzel L-M, Raina A (2016). A phase-field model for chemo-mechanical induced fracture in lithium-ion battery electrode particles. Int J Numer Methods Eng.

[CR44] Mielke A, Roubíček T (2006). Rate-independent damage process in nonlinear elasticity. Math Models Methods Appl Sci.

[CR45] Mussa FF, Horton JD, Moridzadeh R, Nicholson J, Trimarichi S, Eagle KA (2016). Acute aortic dissection and intramural hematoma: a systematic review. J Am Med Assoc.

[CR46] Noble C, van der Sluis O, Voncken RMJ, Burke O, Franklin SE, Lewis R, Taylor ZA (2017). Simulation of arterial dissection by a penetrating external body using cohesive zone modelling. J Mech Behav Biomed Mater.

[CR47] Ogden RW (1997). Non-linear elastic deformations.

[CR48] Ottani V, Raspanti M, Ruggeri A (2001). Collagen structure and functional implications. Micron.

[CR49] Pasta S, Phillippi JA, Gleason TG, Vorp DA (2012). Effect of aneurysm on the mechanical dissection properties of the human ascending thoracic aorta. J Thorac Cardiovasc Surg.

[CR50] Qiao A, Yin W, Chu B (2015). Numerical simulation of fluid–structure interaction in bypassed DeBakey III aortic dissection. Comput Methods Biomech Biomed Eng.

[CR51] Rajagopal K, Bridges C, Rajagopal KR (2007). Towards an understanding of the mechanics underlying aortic dissection. Biomech Model Mechanobiol.

[CR52] Roach MR, Burton AC (1957). The reason for the shape of the distensibility curves of arteries. Can J Biochem Physiol.

[CR53] Roach MR, Song SH (1994). Variations in strength of the porcine aorta as a function of location. Clin Invest Med.

[CR54] Roccabianca S, Ateshian GA, Humphrey JD (2014). Biomechanical roles of medial pooling of glycosaminoglycans in thoracic aortic dissection. Biomech Model Mechanobiol.

[CR55] Ross MH, Pawlina W (2011). Histology: a text and atlas: with correlated cell and molecular biology.

[CR56] Schriefl AJ, Zeindlinger G, Pierce DM, Regitnig P, Holzapfel GA (2012). Determination of the layer-specific distributed collagen fiber orientations in human thoracic and abdominal aortas and common iliac arteries. J R Soc Interface.

[CR57] Schriefl AJ, Schmidt T, Balzani D, Sommer G, Holzapfel GA (2015). Selective enzymatic removal of elastin and collagen from human abdominal aortas: uniaxial mechanical response and constitutive modeling. Acta Biomater.

[CR58] Schulze-Bauer CAJ, Mörth C, Holzapfel GA (2003). Passive biaxial mechanical response of aged human iliac arteries. J Biomech Eng.

[CR59] Sommer G, Gasser TC, Regitnig P, Auer M, Holzapfel GA (2008). Dissection properties of the human aortic media: an experimental study. J Biomech Eng.

[CR60] Sommer G, Sherifova S, Oberwalder PJ, Dapunt OE, Ursomanno PA, DeAnda A, Griffith BE, Holzapfel GA (2016). Mechanical strength of aneurysmatic and dissected human thoracic aortas at different shear loading modes. J Biomech.

[CR61] Svensson RB, Mulder H, Kovanen V, Magnusson SP (2013). Fracture mechanics of collagen fibrils: influence of natural cross-links. Biophys J.

[CR62] Takei A, Roman B, Bico J, Hamm E, Melo F (2013). Forbidden directions for the fracture of thin anisotropic sheets: an analogy with the Wulff plot. Phys Rev Lett.

[CR63] Tam ASM, Sapp MC, Roach MR (1998). The effect of tear depth on the propagation of aortic dissections in isolated porcine thoracic aorta. J Biomech.

[CR64] Teichtmeister S, Kienle D, Aldakheel F, Keip M-A (2017). Phase-field modeling of fracture in anisotropic brittle solids. Int J Non Linear Mech.

[CR65] Thubrikar MJ, Agali P, Robicsek F (1999). Wall stress as a possible mechanism for the development of transverse intimal tears in aortic dissections. J Med Eng Technol.

[CR66] Tong J, Sommer G, Regitnig P, Holzapfel GA (2011). Dissection properties and mechanical strength of tissue components in human carotid bifurcations. Ann Biomed Eng.

[CR67] Tong J, Cohnert T, Regitnig P, Kohlbacher J, Birner-Gruenberger R, Schriefl AJ, Sommer G, Holzapfel GA (2014). Variations of dissection properties and mass fractions with thrombus age in human abdominal aortic aneurysms. J Biomech.

[CR68] Tsamis A, Krawiec JT, Vorp DA (2013). Elastin and collagen fibre microstructure of the human aorta in ageing and disease: a review. J R Soc Interface.

[CR69] Tse KM, Chiu P, Lee HP, Ho P (2011). Investigation of hemodynamics in the development of dissecting aneurysm within patient-specific dissecting aneurismal aortas using computational fluid dynamics (CFD) simulations. J Biomech.

[CR70] Wang Y, Johnson JA, Spinale FG, Sutton MA, Lessner SM (2014). Quantitative measurement of dissection resistance in intimal and medial layers of human coronary arteries. Exp Mech.

[CR71] Wang L, Zhu J, Samady H, Monoly D, Zheng J, Guo X, Maehara A, Yang C, Ma G, Mintz GS, Tang D (2017). Effects of residual stress, axial stretch, and circumferential shrinkage on coronary plaque stress and strain calculations: a modeling study using IVUS-based near-idealized geometries. J Biomech Eng.

[CR72] Wang L, Hill NA, Roper SM, Luo X (2018). Modelling peeling- and pressure-driven propagation of arterial dissection. J Eng Math.

